# Evaluating multiple stability methods to screen bread wheat genotypes (F7 generation) under drought-stressed environments

**DOI:** 10.7717/peerj.20505

**Published:** 2026-02-23

**Authors:** Armin Saed-Moucheshi, Shahryar Sasani, Farshad Bakhtiar, Davod Roodi, Shokoofeh Sarikhani Khorami

**Affiliations:** 1 Crop and Horticultural Sciences Research Department, Kermanshah Agriculture and Natural Resources Research and Education Center, Kermaanshah, Iran; 2 Crop and Horticultural Science Research Department, Kermanshah Agricultural and Natural Resources Research and Education Center, AREEO, Kermaanshah, Iran; 3 Seed and Plant Improvement Institute (SPII), Agriculture Research Education and Extension Organization, Karaj, Iran; 4 Agricultural Research, Agriculture Research Education and Extension Organization, Neishabour, Iran; 5 Seed and Plant Improvement Research Department, Fars Agricultural and Natural Resources Research and Education Center, AREEO, Zarghan, Iran, Gorgan University Of Agricultural Sciences And Natural Resources, Zarghan, Iran

**Keywords:** Programming in R language, Multi-environment stability, Novel stability models, CIMMYT wheat breeding, Heatmap-Clustering

## Abstract

Wheat, as a staple food crop, faces productivity challenges under diverse environmental conditions, particularly in semi-arid regions. Enhancing genotype performance and stability across multiple environments is essential for sustainable wheat production and food security. This study aimed to evaluate the grain yield performance and stability of 165 F_7_ wheat genotypes, along with four check cultivars, across four agro-ecologically distinct environments: Karaj, Zarghan, Kermanshah, and Nishapur, with the latter representing drought-prone conditions. The genetic materials used were derived from both local germplasm and International Maize and Wheat Improvement Center; “Centro Internacional de Mejoramiento de Maíz y Trigo” (CIMMYT) sources, ensuring a wide genetic base and reflecting the benefits of international collaboration in crop improvement. A combination of univariate and multivariate stability analyses, including combined-analysis of variance (ANOVA), the additive main effects and multiplicative interaction (AMMI) and *genotype and genotype* × *environment interaction (GGE)* model heatmap-clustering, and correlation plots, was conducted to explore the genotype-by-environment interactions (GEI). An R-based script was developed and introduced to facilitate reproducible and efficient computation of these stability models (Supplementary Materials). The ANOVA revealed significant genotype-by-environment interaction (GEI) effects, highlighting the necessity for robust statistical approaches. AMMI analysis showed that the first two interaction principal components (IPCA1 and IPCA2) accounted for over 83% of the interaction variance, effectively capturing differential genotype responses. Genotypes G48, G46, and G122 were consistently high-yielding and stable across all environments. Local genotypes demonstrated broad adaptability, while CIMMYT-derived lines exhibited superior performance under drought conditions, especially in Nishapur. This study demonstrates the utility of integrating classical and modern statistical tools for selecting high-performing and stable wheat genotypes, providing valuable insights for breeders targeting multi-environment adaptation and drought tolerance. The identified genotypes offer promising candidates for additional breeding programs aimed at improving yield stability, which can be considered in future studies focusing on validating the genotypes.

## Introduction

Wheat (Triticum aestivum L.) is a cornerstone of global agriculture, serving as a primary food source for a significant portion of the world’s population. As of 2023, the global cultivation area for wheat is estimated at approximately 220 million hectares, with Asia contributing a substantial share (FAO, 2023). In Iran, wheat is cultivated on over 6 million hectares of land, underscoring its strategic role in national food security and rural livelihoods (AREEO, 2024). This importance is further magnified in Iran’s arid and semi-arid zones, where wheat production is critically vulnerable to environmental stressors, especially drought (Aliakbari et al., 2013). According to recent statistics, the average wheat grain yield in Iran during the 2022/2023 growing season was about 1.0 t/ha under rain-fed conditions and 3.0 t/ha under irrigation; significantly below the attainable potential of 5–6 t/ha in optimal environments (AREEO, 2024). This persistent yield gap is attributed to multiple factors, including the absence of high-yielding and environmentally stable cultivars, along with the presence of biotic (Jaggard, Qi & Ober, 2010; Connor, 2022) and abiotic stresses (Rockström & Falkenmark, 2000; Tian et al., 2021) that compromises productivity across regions.

Among abiotic stressors, drought remains the most prevalent and destructive factor affecting global wheat yields. It is estimated that drought stress alone can reduce wheat production by up to 50% in vulnerable regions (Ahmad et al., 2018). Drought affects wheat physiology at multiple levels, impairing photosynthesis, disrupting water and nutrient uptake, and causing oxidative damage to cellular structures (Saed-Moucheshi et al., 2019). Wheat genotypes vary widely in their ability to tolerate drought, employing mechanisms such as osmotic adjustment, increased root depth, delayed senescence, and enhanced antioxidant activity (Sohrabi et al., 2024). These adaptive traits are often polygenic and interact strongly with environmental conditions, making the identification and selection of drought-tolerant genotypes a complex but critical breeding objective (Saed-Moucheshi et al., 2021). Incorporating diverse genetic resources, including landraces and international germplasm such as those from CIMMYT, is essential for developing resilient cultivars (Saed-Moucheshi, Babaei & Ansarshourijeh, 2024). Such efforts contribute not only to improved crop performance but also to long-term agricultural sustainability and food security under climate uncertainty (Saed-Moucheshi et al., 2023).

To address yield instability under environmental variation, particularly drought stress, multi-environment trials (METs) serve as indispensable tools in modern plant breeding. METs allow breeders to assess genotype performance across diverse agro-ecological zones and to identify broadly adapted or specifically adapted genotypes. Each year, global breeding programs conduct extensive METs across major crops, enabling robust genotype comparisons based on multiple phenotypic traits (Vosough, Ghouchani & Saed-Moucheshi, 2019). However, interpreting genotype-by-environment interactions (GEI) within METs necessitates advanced statistical models. Stability indices classified into univariate (parametric and non-parametric) and multivariate types are routinely used for this purpose (Saed-Moucheshi et al., 2013). Parametric measures like regression coefficients and coefficients of variation offer intuitive insights into yield performance trends, while non-parametric rank-based indices provide robustness against data distribution assumptions. Among multivariate approaches, principal component analysis (PCA)-based techniques, such as the additive main effects and multiplicative interaction (AMMI) model and the genotype and genotype × environment interaction (GGE) biplot, have become standard tools in GEI analysis for identifying superior and stable genotypes (Saed-Moucheshi, Babaei & Ansarshourijeh, 2024).

Recent studies have highlighted the efficacy of combining univariate and multivariate stability indices in assessing wheat genotypes under drought stress conditions. For example, Saed-Moucheshi et al. (2023) highlighted the effectiveness of the GGE biplot in identifying genotypes with favorable drought responses, while other works emphasize the value of AMMI-based clustering and visual heatmaps for ranking genotypes under stress (Derbew et al., 2024). Despite these advances, there remains a notable gap in the literature regarding the integration of stability indices with modern computational tools for drought tolerance screening. In particular, few studies have comprehensively applied both univariate and multivariate models. Some studies have considered the environmental clustering to evaluate genotypes derived from diverse genetic backgrounds (*e.g.*, local × CIMMYT) within the context of drought-prone and variable Iranian agro-ecologies (Regmi et al., 2021).

Accordingly, the present study aims to bridge this gap by evaluating 165 F_7_ wheat genotypes and four check cultivars across four diverse environments in Iran using an integrated set of stability indices. By combining classical and multivariate statistical tools—along with the development of an R-based computational pipeline—this research seeks to identify genotypes with high grain yield, broad adaptability, and superior performance under drought stress. The findings are expected to support breeding programs targeting drought resilience and yield stability, thereby contributing to sustainable wheat production under increasingly variable climatic conditions.

## Materials and Methods

### Plant materials and experimental populations

A total of 165 F_7_ wheat lines alongside four check cultivars were evaluated in this study. The F_7_ lines were derived from two sources: (1) crosses conducted within the Agricultural Research, Education and Extension Organization (AREEO) advanced to F_6_, following selection of promising families in national breeding programs; and (2) international germplasm from CIMMYT, specifically from the Semi-Arid Wheat Screening Nursery (SAWSN) set (https://gender.cgiar.org/publications/34th-semi-arid-wheat-screening-nursery-34th-sawsn-cycle-2016) and Semi-Arid Wheat Yield Trial (SAWYT) set (https://datahub.moa.gov.et/), which focus on drought-adapted germplasm for wheat mega environments. Pedigree details for all entries are provided in Table S1.

#### Check cultivars (control)

The four check cultivars, including Amin, Danesh, Farin, and Torabi, are newly released cultivars in AREEO’s breeding programs. Additionally, Amin is a recently released local irrigated cultivar developed by AREEO, which exhibits superior adaptation; however, detailed published agronomic data remain limited. Danesh (Wheat line M-96-13), introduced *via* CIMMYT’s 47th International Bread Wheat Screening Nursery (IBWSN), was released in Iran in 2021 for its high yield potential, rust resistance, lodging resistance, and excellent bread-making quality. Farin, derived from CIMMYT germplasm (line M-94-15), underwent multiple national trials before its release in 2019 due to superior yield performance under both normal and stressed conditions, plus disease resistance and baking quality. Torabi (line M-92-20) originated from the 28th SAWSN trial. It exhibited strong terminal drought tolerance, high yields under stress, rust resistance, early maturity, and bread quality. In addition, this line was promoted for farmer-level trials after multi-environment testing (Saed-Moucheshi, Babaei & Ansarshourijeh, 2024). These check cultivars were selected for their high performance and their drought tolerance. These cultivars are locally approved and provide relevant benchmarks for comparison with new lines.

### Experimental sites and layout

Field trials were conducted during the 2022–2023 cropping season at four locations in Iran: Karaj, Zarghan, Kermanshah, and Nishapur (Nishapur), which represent diverse climatic zones, from temperate to semi-arid. The experimental design was an augmented design, with the four check cultivars randomly replicated in quadruplicate across incomplete blocks containing the 165 unreplicated F_7_ lines, following standard augmented designs in plant breeding to control for spatial variability. It must be stated that the replication of each genotype in the augmented design is equal to 1. In our study there is no need for higher replication to calculate the stress indices, according to their formulas.

#### Drought stress treatment and environmental monitoring

In Nishapur, the trial was managed as a drought-stress environment: traditional local irrigation was suspended at the flowering stage to induce terminal drought stress. In the other sites, irrigation followed local practices, maintaining soil moisture at ∼75–80% of field capacity until physiological maturity. Soil moisture monitoring was conducted *via* gravimetric sampling and Time Domain Reflectrometry (TDR) probes at 10-day intervals to confirm drought conditions onset and dynamics. Meteorological data, including rainfall, temperature, humidity, and evapotranspiration, were recorded at each site using automated weather stations. Soil properties (texture, organic matter, pH, available nutrients) were determined according to standard AREEO protocols (Table S2).

### Agronomic practices

Plots (6 m^2^ each) were sown at a density of 350 seeds/m^2^ using localized sowing machinery. At sowing, fertilization included urea (N) at 75 kg/ha and diammonium phosphate (DAP) at 50 kg/ha. A top dressing of urea at 100 kg/ha and an additional 50 kg/ha of N were applied at stem elongation stage (Zadoks 31 32). All sites followed uniform weed, disease, and pest management protocols in line with AREEO guidelines. Finally, at physiological maturity, all plants in each plot were manually harvested. Grain yield was recorded per plot (kg per 6 m^2^) and standardized to kg/m^2^ for statistical analysis.

### Statistical analysis

Yield data were analyzed using ANOVA for augmented designs, treating checks as replicated controls and F_7_ lines as unreplicated treatments. Genotype × environment interactions (GEI) were examined *via* combined ANOVA across sites. Stability and adaptability were assessed using both univariate (parametric: regression coefficient, coefficient of variation; non-parametric: rank-based stability) and multivariate analyses, including the additive main effects and multiplicative interaction (AMMI) and GGE biplot models. For the univariate stability indices, a special code was transcribed as an R-Script (applicable in all versions of the R language) by the first author. This code has been tested on several datasets from different experiments, and the results from this code were equal to, while more precise than the results from the hand calculation. This code has never been written or used in any other study. This code can be downloaded from the Supplementary Materials or the git hub address (https://github.com/ArminSaed/PBTolindex). Other analyses, such as heatmaps, cluster dendrograms, and correlation plots, were generated to visualize genotype performance patterns using the R language.

Finally, the genotypes were sorted according to their rank obtained from each index (both univariate and multivariate methods) based on the method described in Saed-Moucheshi, Babaei & Ansarshourijeh (2024) for all environments. The univariate and multivariate indices used in this study are available in the packages that the authors wrote are as follows:

#### Environmental Variance (S^2^)


\begin{eqnarray*}{S}_{i}^{2}= \frac{1}{n-1} \sum _{j=1}^{n}({X}_{ij}-{\bar {X}}_{i})^{2} \end{eqnarray*}

*X*_*ij*_ is the yield of genotype *i* in environment *j*.

${\bar {X}}_{i}$ is the mean yield of genotype *i* across all environments.

*n*: is the number of environments.

#### Coefficient of Variation (CV)


\begin{eqnarray*}CV= \left( \frac{{S}_{i}}{{\bar {X}}_{i}} \right) \times 100 \end{eqnarray*}

*S*_*i*_ is the standard deviation of genotype iii across environments.

${\bar {X}}_{i}$ is the mean yield of genotype *i* across all environments.

#### Standard Deviation (SD)


\begin{eqnarray*}{S}_{i}=\sqrt{ \frac{1}{n-1} \sum _{j=1}^{n}({X}_{ij}-{\bar {X}}_{i})^{2}} \end{eqnarray*}

*X*_*ij*_ is the yield of genotype *i* in environment *j*.

${\bar {X}}_{i}$ is the mean yield of genotype *i* across all environments.

*n*: is the number of environments.

#### Wricke’s Ecovalence (W^2^)


\begin{eqnarray*}{W}_{i}^{2}=\sum _{j=1}^{n}({X}_{ij}-{\bar {X}}_{i}-{\bar {X}}_{j}-\bar {X})^{2} \end{eqnarray*}

${\bar {X}}_{ij}$ is the yield of genotype *i* in environment *j*.

${\bar {X}}_{i}$ is the mean yield of genotype *i* across all environments.

${\bar {X}}_{j}$ is the mean yield of environment *j* across all genotypes.

$\bar {X}$ is the mrand mean yield across all genotypes and environments.

#### Shukla’s stability variance (*σ*^2^)


\begin{eqnarray*}{\delta }_{i}^{2}= \frac{\sum _{j=1}^{n}({Y}_{ij}-{Y}_{i.}-{Y}_{.j}-{Y}_{..})^{2}}{(n-1)} \end{eqnarray*}

*Y*_*ij*_ is the mean performance of genotype iii in environment *j*.

*Y*_*i*_. is the mean of genotype iii across all environments.

*Y*_.*j*_ is the mean of all genotypes in environment *j*.

*Y*_.._ is the grand mean across all genotypes and environments.

*n* is the number of environments.

#### Regression coefficient (b)


\begin{eqnarray*}{b}_{i}= \frac{\sum _{j=1}^{n} \left( {X}_{ij}-{\bar {X}}_{i} \right) ({E}_{i}-\bar {E})}{\sum _{j=1}^{n}{ \left( {E}_{i}-{\bar {E}}_{i} \right) }^{2}} \end{eqnarray*}

*X*_*ij*_ is the yield of genotype *i* in environment *j*.

${\bar {X}}_{i}$ is the mean yield of genotype *i* across all environments.

*E*_*j*_ is the environmental index for environment *j* (mean yield across all genotypes in environment *j*).

$\bar {E}$ is the mean of environmental indices.

#### Eberhart and russell’s deviation from regression (Sd^2^i)


\begin{eqnarray*}s{d}_{i}^{2}= \frac{\sum _{j=1}^{n}({X}_{ij}-{\bar {X}}_{i}-{b}_{i}({E}_{i}-{\bar {E}}_{i}))^{2}}{(n-2)} \end{eqnarray*}

*X*_*ij*_ is the yield of genotype *i* in environment *j*.

${\bar {X}}_{i}$ is the mean yield of genotype *i* across all environments.

*E*_*j*_ is the environmental index for environment *j* (mean yield across all genotypes in environment *j*).

$\bar {E}$ is the mean of environmental indices.

#### Coefficient of determination (R^2^)


\begin{eqnarray*}{R}_{i}^{2}=1- \frac{\sum _{j=1}^{n}{ \left( {X}_{ij}-{\hat {X}}_{ij} \right) }^{2}}{\sum _{j=1}^{n}{ \left( {X}_{ij}-{\bar {X}}_{i} \right) }^{2}} \end{eqnarray*}

*X*_*ij*_ is the yield of genotype *i* in environment *j*.

${\bar {X}}_{i}$ is the mean yield of genotype *i* across all environments.

*n*: is the number of environments.

${\hat {X}}_{ij}$ is the predicted value of each genotype across each environment by the regression model.

#### Multivariate stability analyses

a. Additive main effects and multiplicative interaction (AMMI) model:

\begin{eqnarray*}{Y}_{ij}=\mu +{G}_{i}+{E}_{j}+\sum _{k=1}^{n}{\lambda }_{k}{\gamma }_{ik}{\delta }_{jk}+{\rho }_{ij} \end{eqnarray*}

*Y*_*ij*_ is the observed yield of genotype *i* in environment *j*.

µis the grand mean of all genotypes in all environments.

*G*_*i*_ is the genotype *i* effect.

*E*_*j*_ is the environment *j* effect.

*λ*_*k*_ is the singular value for principal component *k*.

*γ*_*ik*_ is the genotype *i* score for principal component *k*.

*δ*_*jk*_ is the environment *j* score for principal component *k*.

*ρ*_*ij*_ is the residual term.

b. Genotype and genotype × environment interaction (GGE) biplot:

\begin{eqnarray*}{Y}_{ij}=\mu +{E}_{j}+\sum _{k=1}^{n}{\lambda }_{k}{\xi }_{ik}{\eta }_{jk}+{\epsilon }_{ij} \end{eqnarray*}

*Y*_*ij*_ is the performance of genotype *i* in environment *j*.

µis the grand mean.

*E*_*j*_ is the environment *j* effect.

*λ*_*k*_ is the singular value for the principal component *k*.

*ξ*_*ik*_ is the eigenvector of genotype *i* for principal component *k*.

*η*_*jk*_ is the eigenvector of environment *j* for principal component *k*.

*ϵ*_*ij*_ is the residual associated with genotype *i* in environment *j*.

Considering each univariate index as well as their ranks is very hard to perform. Also, the results of the univariate indices are not always in line with one another. Therefore, the authors have solved this problem by suggesting the use of all rank results together. The new novel method combines the values of each genotype provided by all univariate indices into one final rank by calculating the harmonic average of the values of all ranks. It should be noted that the ranks were calculated based on the values of indices, so that the greater the value, the lower the ranking value. For calculating the new method (Stability Identity Document (SID)) uses the inverse values of the indices that have a negative correlation with the grain yield. This described method is also included in the R-Script provided as Supplementary Materials by executing the command “sid()” in “Supplementary_Stbidx_Code_AMMI_GGE”. This command can also provide the rank of each genotype based on the SID index that combines all stability indices under the name “Ranked Sustainability ID (RSID)”.

The correlation analysis was performed by R language in the RStudio environment based on the Pearson method and it turned into a heatmap under the name of correlation plot. This correlation plot was used for presenting the correlations and the strength of the associations among all indices. For this assessment “agricolae” and “Pheatmap” packages from R libraries were used. The formula of Pearson correlation is as follows:

\begin{eqnarray*}r= \frac{\sum _{i=1}^{n} \left( {X}_{i}-{\bar {X}}_{i} \right) ({Y}_{i}-{\bar {Y}}_{i})}{\sqrt{\sum _{i=1}^{n}{ \left( {X}_{i}-{\bar {X}}_{i} \right) }^{2}}\times \sqrt{\sum _{i=1}^{n}{ \left( {Y}_{i}-{\bar {Y}}_{i} \right) }^{2}}} . \end{eqnarray*}

In which *X* and *Y* are signs for each of the indices that are about to be applied for correlation calculations, and *i* is the number of each genotype. $\bar {X}$ and $\bar {Y}$ are the averages of each used index for correlation calculation.

### Mock-experiment example

To illustrate the reproducibility of the pipeline and statistical approach, a simulated dataset (mock experiment) is included in the Supplementary Materials. This example demonstrates data formatting, R-Script, and interpretation of outputs, all following the structure used for actual trial data.

## Results

**Figure 1:**
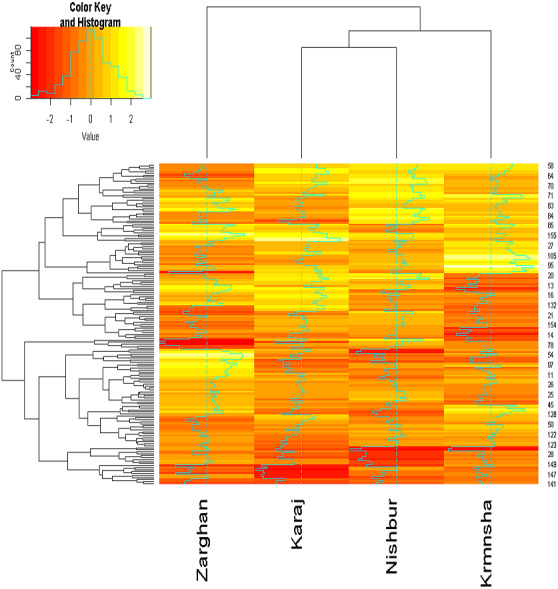
Heatmap and clustering of genotypes and environment according to grain yield.

The analysis of variance (ANOVA) revealed significant differences in grain yield among the wheat genotypes across the tested environments, indicating a substantial genotype-by-environment (G×E) interaction. This result suggests that the yield of genotypes varied depending on the environmental conditions. The mean grain yield across all genotypes and environments was 6.998 tons per hectare, with individual genotype means ranging from 3.111 to 10.877 tons per hectare. This result also indicates that there may be some (or one) genotype performing best (or well) in some environments, while insufficient in other environments. To choose such stable genotypes, the stability analysis based on univariate and multivariate methods was performed. Also, a combination of heatmap and cluster techniques was used to indicate the similarity of genotypes and environments (Fig. 1). This method can be addressed as a novel method of depicting the yield of genotypes under varied environments. Accordingly, the results indicated that Nishapur and Karaj were enclosed as one cluster, while Kermanshah and Zarghan were separately included in two clusters.

### Univariate stability analysis

**Figure 2:**
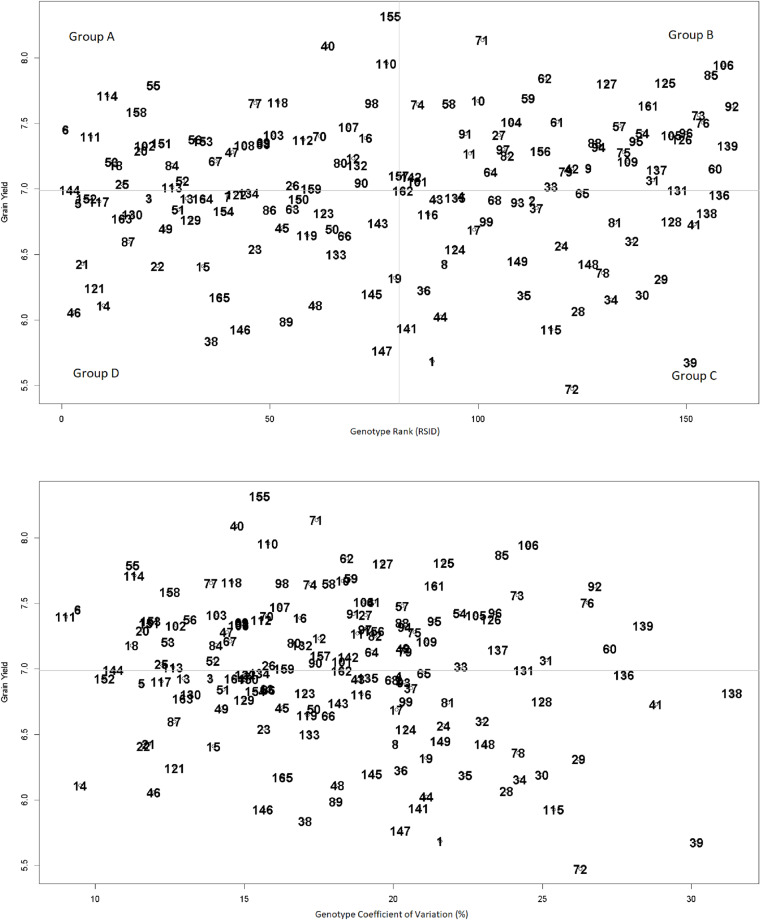
Average grain yield of each genotype versus genotype’s coefficient of variation across all environments (upper panel) and genotype’s rank according to all calculated univariate stability methods (lower panel).

The analysis of genotype stability using univariate stability indices provided valuable insights into the performance consistency of the evaluated genotypes across different environments. The mean performance of the best genotypes varied depending on the stability index used for selection. The environmental variance (S^2^) identified G14 as the most stable genotype. G14 showed a mean yield of 6.109 t ha^−^^1^ (Table S3) and the lowest environmental variance (0.336). Similarly, the coefficient of variation (CV) highlighted G111 as the most stable genotype, with a mean yield of 7.4 t ha^−^^1^ and the lowest CV value (9.018). Based on the results from the CV depicting *versus* grain yield, in Fig. 2A, G6 was closest to G111. Therefore, this genotype can be considered as another proper genotype aside from G111. The regression coefficient (bi) approach indicated that G48 had the best adaptability to altered environments. G48 showed a mean yield of 6.112 t ha^−^^1^ and a regression coefficient closest to 1 (bi = 1.002), suggesting an average response to environmental changes.

The deviation from regression (S^2^di) criterion ranked G153 as the most stable, with a mean yield of 7.367 t ha^−^^1^ and the lowest deviation from regression. this result indicates minimal fluctuation around the predicted response (0.5344) for this genotype. Wricke’s ecovalence (Wi^2^) and Shukla’s stability variance (*σ*i^2^) did not show a significant correlation with yield in the current study. However, Wricke’s ecovalence (Wi^2^) showed that G48 exhibited the least contribution to the genotype-by-environment interaction, with a mean yield of 6.112 t ha^−^^1^ and the lowest Wi^2^ value (0.026). Finally, Shukla’s stability variance (*σ*i^2^) indicated that G48 was the most stable, with a mean yield of 6.112 t ha^−^^1^ and the lowest *σ*i^2^, implying consistent performance across environments (0.00015).

Since the results of each univariate analysis were not the same, each genotype based on each index was ranked, and the results are presented in Table S4. Accordingly, the rank 1 represents the best performance genotypes; therefore, lower-ranked genotypes indicate higher performance. For the univariate methods, including S2, CV, W2, sig2, b, sd, and R^2^ genotypes G13, G111, G48, G48, G48, G153, G19 obtained rank 1, respectively. Therefore, we calculated the mean rank for each genotype based on all indices that have not been applied in previous studies. The result showed that the mean ranks of genotypes G6, G144, G48, G46, G5, G21, G153, G111, G121, G117, G14, and G114 were between 33 to 36. Additionally, the worst genotypes with rank values between 137 to 151 were G92, G139, G106, G136, G60, G85, G138, G76, G73, and G41.

The scatter plot of the grand mean yield of each genotype *versus* the final ranked genotypes based on the described method in the Materials and Methods section, which is mentioned as the RSID method is presented in Fig. 2B. It must be noted that such a scatter plot has not been used in previous studies, to our knowledge. Based on this method in Fig. 2B, the genotypes can be divided into four groups within the section that are separated by average lines. Accordingly, the most proper genotypes are those included within section one with yield values higher than the grand mean and the rank values lower than the mean rank. Genotypes number 155 and 11 have RSID values roughly equal to the mean value and are included in Group A. On the other hand, genotypes 55, 144, and 158 showed high values for yield grand mean and low values for RSID.

**Figure 3:**
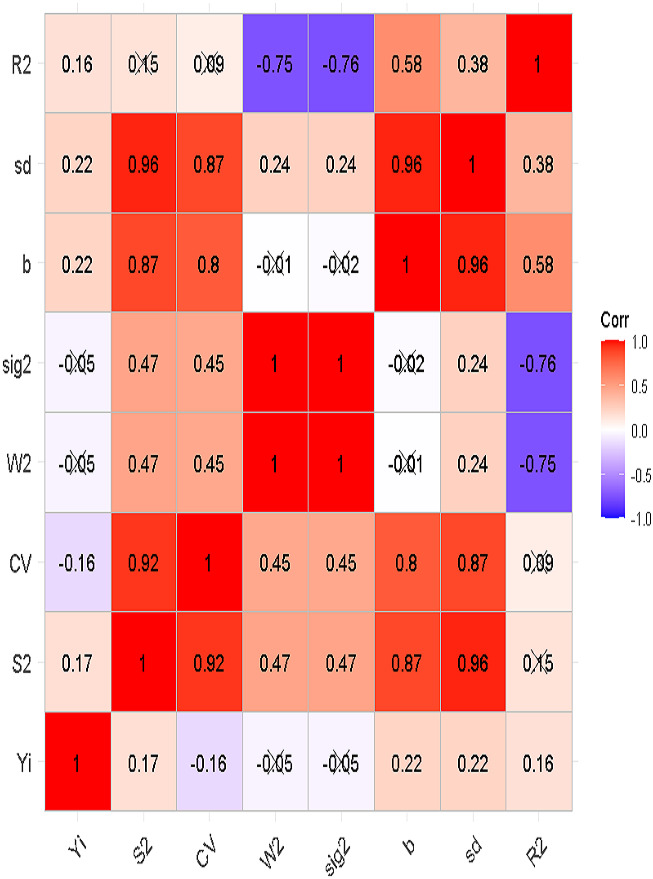
Correlation plot presenting the association between the univariate stability methods calculated for 165 genotypes across four environments. Yi, Grain yield; S2, Environmental Variance; CV, Environmental Coefficient of Variation; W2, Shukla Environmental Variance; sig2, Significance of Regression Model; b, Coefficient of Regression Lines; sd, Standard Deviation or Error Term in Regression Model; R2, Coefficient of Determination of the regression model.

A comparison of the different univariate stability indices revealed the effectiveness in identifying high-yielding and stable genotypes. To screen high-performance univariate indices, the correlation between each pair of univariate indices was calculated and depicted in a heatmap, as shown in Fig. 3. The results showed that some indices, such as the regression coefficient (bi), are in favor of genotypes with average adaptability. Other indices, like the deviation from regression (S^2^di) and Shukla’s stability variance (*σ*i^2^), are emphasizing stability with minimal environmental fluctuation. The environmental variance (S^2^) and coefficient of variation (CV) were more sensitive to genotypic fluctuations, often selecting moderate yields but consistent performance genotypes. While Shukla’s stability variance (*σ*i^2^) and Wricke’s ecovalence (Wi^2^) tried to point out the genotypes with high yield and high stability. However, the results of correlation showed that these indices had a negative, but not significant, correlation with grain yield. The highest positive and significant correlations with grain yield belonged to the regression coefficient (bi) and deviation from regression (S^2^di), equal to *r* = 0.22.

### Multivariate stability analysis

**Figure 4:**
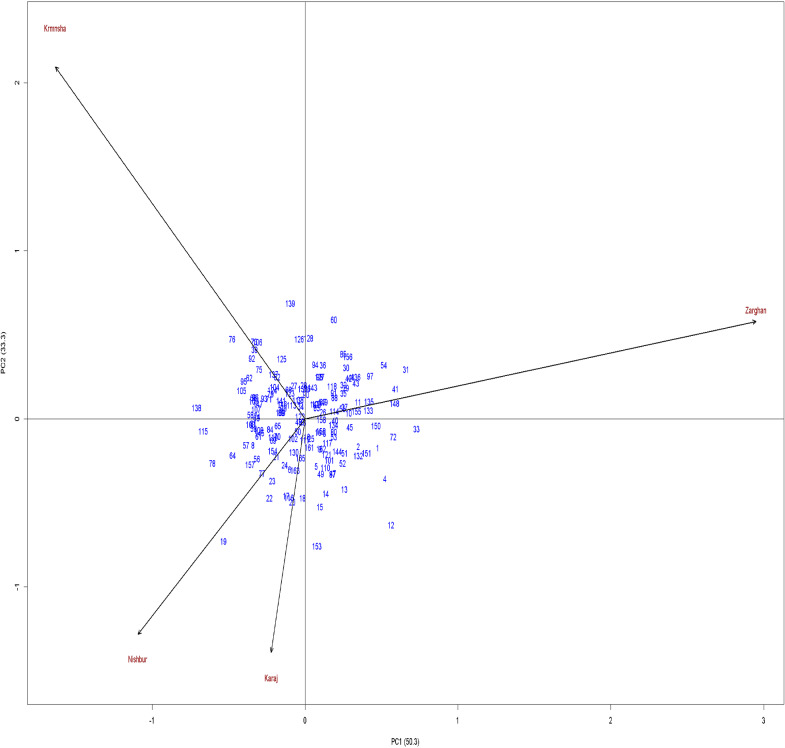
Biplot based on additive main effects plus interaction effects (AMMI) for genotypes and environments.

The additive main effects and multiplicative interaction (AMMI) analysis was employed to further consider the G×E interaction. The AMMI model revealed that the first two interaction principal component axes (IPCA1 and IPCA2) accounted for 50.3% and 33.3% of the interaction sum of squares, respectively, explaining a substantial portion of the G×E variability (Fig. 4). The combined results of the AMMI model and ANOVA for grain yield are presented in Table 1. This table also shows that the first two components were able to explain over 83 of the variability. Genotypes G56, G62, G172, G122, G48, G46, G158, and G40 had an IPCA1 score close to zero, indicating high stability based on IPCA1. However, genotype G139 exhibited a large positive IPCA1 score, suggesting specific adaptation to certain environments. G18, G65, G161, G111, G10, G48, G46, G122, G90, G28, and G86 were the genotypes closest to zero line of IPCA2, while G138, G115, and G33 were the farthest from this line. Combining both IPCA1 and IPCA2, G122, G48, and G46 were the genotypes closest to the center of the IPCA plot (AMMI biplot) with the lowest values of IPCA1 and IPCA2. Similar to the heatmap-cluster result, Karaj and Nishapur environments were positioned in the same sectors of the biplot, while they were significantly distanced from Zarghan and Kermanshah. Also, Kermanshah and Zarghan were placed in separate sections of the biplot.

**Table 1:** Combined analysis of variance (CoANOVA) based on AMMI model for the main and interaction sources of variation along with principal components assessed from the interaction effect of environment by genotype.

Source	Degree freedom	Sum sq	Mean sq	F-Value	Probability	Sig Level	Accumulated (%)
Environment: Env	3	805.9	268.663	73.9543	0.000585	***	NaN
Replication (ENV)	4	14.53	3.633	5.6039	0.00882	**	NaN
Genotype: Gen	160	197.5	1.234	1.9043	0.103812	ns	NaN
Env × Gen	480	323.7	0.674	1.0402	0.514848	ns	NaN
PC1	162	317.9	1.96	3.03	0.0172	*	50.3
PC2	160	210.3	1.31	2.03	0.0831	ns	83.7
PC3	158	103.2	0.65	1.01	0.5392	ns	100
Sum	480	323.7	0.67	NaN	NaN	NaN	100
Residuals	12	7.78	0.648	NaN	NaN	NaN	NaN
Coefficient of Variation (%)			11.50501				

**Notes.**

Sq Squares sig Significance PC Principal Component *** Significant at 0.001 level ** Significant at 0.01 level * Significant at 0.05 level ns Not Significant NaN not Available

Replication (Environment) was computed based on the control cultivars (Danesh, Amin, Farin, Torabi) repeated in all environments. Environment, Genotype, and Environment by Genotype are assessed based on all environments and all genotypes excluding the controls.

**Figure 5:**
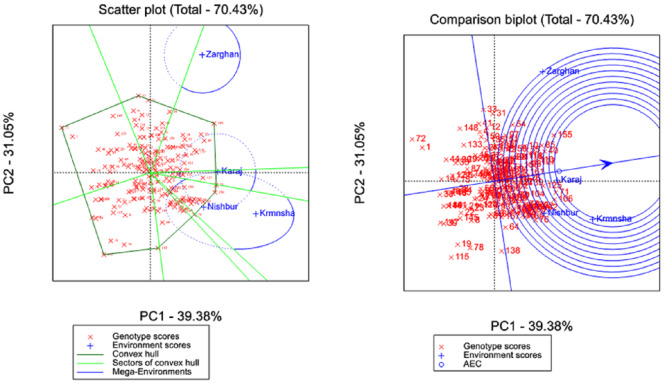
Biplot of first-two components estimated by genotype plus genotype by environment method.

The genotype and genotype × environment (GGE) biplot analysis in Fig. 5 provided a visual representation of the genotypic performance and stability. Figure S1 showed that the first PCA and second PCA of the GGE biplot were able to capture more than 75% of the variability among genotypes. In addition, the first PCA mainly contained the genotype effect, while the second PCA mostly contained the genotype-by-environment effect. The GGE biplot identified several “mega-environments”, each with a specific set of high-performing genotypes. GGE biplot divided the result into seven sectors through which Kermanshah and Nishapur were placed in the same sector, separated from Karaj and Zarghan. However, Karaj and Nishapur were closest to the center, indicating low variability among the data from these two environments. The most distant environment from the center of the GGE biplot was Zarghan. For Zarghan and Kermanshah, which were placed far from the center, the genotypes were not near enough to be favored for these environments. However, G106 and G76 were closer to the Kermanshah environment, and G155, G31, and G33 were closer to Zarghan compared to other genotypes. G125 and G76 were the closest genotypes to Karaj and Nishapur, respectively. Genotypes G1 and G72 were the wheat genotypes with the highest distances from all tested environments, and from the center of the GGE biplot (Fig. S2). All environments had positive values for the first biplot PCA, while both negative and positive values for the second biplot PCA (Fig. S2). Therefore, genotypes with higher positive values of the first PCA and values close to zero for the second PCA can be identified as the favorite genotypes for all environments. Accordingly, G125, G39, G71, G161, G48, and G46 were the genotype favorites for all environments.

## Discussion

Evaluating wheat genotypes across diverse environments is crucial for identifying high-yielding and stable cultivars under changing conditions. This study was carried out to investigate the performance and stability of 165 F7 wheat genotypes and four check cultivars across four distinct environments, including a drought-stressed condition. Stability was assessed using univariate (CV, SD, R^2^, W^2^, *σ*^2^, b) and multivariate (AMMI, GGE) stability indices. The significant genotype × environment (G×E) interaction observed highlights the need for multiple complementary statistical methods to fully elucidate genotype performance (Cooper et al., 2022). This integrated approach, combining univariate and multivariate analyses, has become increasingly recognized as essential for robust genotype evaluation (Biradar et al., 2024).

The significant G×E interaction indicates that genotype performance varied substantially among tested environments. The univariate stability indices used in this study can be categorized into three groups. The first group of indices, such as the regression coefficient (bi), favors genotypes with average adaptability. The second group, including environmental variance (S^2^) and coefficient of variation (CV), is more sensitive to genotypic variations across environments and moderate yields, but consistent performance, genotypes across multiple environments. The third group of indices, such as deviation from regression (S^2^di) and Shukla’s stability variance (*σ*i^2^), emphasized stability with minimal environmental variations and fluctuation. According to the first group, genotype G48, according to the second group, G14, G6, and G111, and according to the third group, G48 and G13, were among the most favorite genotypes. It should be noted that Wricke’s ecovalence (Wi^2^) and Shukla’s stability variance (*σ*i^2^) correlations with grain yield were not statistically significant. However, some studies (Nesa, Das & Sagor, 2024; Güngör et al., 2024) have indicated their application as proper indices for screening. Therefore, the results of these indices were considered in this study. Additionally, the result of RSID method showed that genotypes 155 and 110 have RSID values roughly equal to the mean value and are included in Group A. While genotypes 55, 144, 158, 111, and six showed high values for yield grand mean and low values for RSID. These genotypes could be considered as the most suitable genotypes having both proper stability against environmental changes and high grain yield.

Regarding the performance of univariate indices in this study, while Shukla’s stability variance (*σ*i^2^) and Wricke’s ecovalence (Wi^2^) aimed to identify genotypes with high stability. The results of the correlation showed a negative, but not statistically significant, correlation with grain yield. While negative correlations with yield were observed for Shukla and Wrick indices in the current study. Some previous studies (Linus et al., 2023; Rezaizad et al., 2025) presented these indices as the most efficient indices for screening favorable, stable genotypes highly correlated with grain yield. Therefore, our results cannot verify most previous studies result regarding the Wrick and Shukla indices as the most reliable to identify high-yield genotypes. The negative correlation of these indices might be due to their focus on variability and genotype fluctuation across environments. Meanwhile, the highest positive and significant correlations with grain yield were observed in the regression coefficient (bi) and deviation from regression (S^2^di). While useful for identifying genotypes with relatively stable performance, these indices have limitations in fully capturing the complexity of G×E interactions (Mumford et al., 2023). Studies comparing stability indices have shown that while CV and SD can identify general stability, they may not adequately distinguish between general and specific adaptability (Saed-Moucheshi, Babaei & Ansarshourijeh, 2024). Our findings corroborate this, demonstrating that univariate indices alone were insufficient to explain all variations in genotype stability, highlighting the need for multivariate approaches.

AMMI analysis revealed that the firsttwo principal components captured the majority of the interaction variance. This is consistent with the previous studies demonstrating AMMI’s effectiveness in dissecting genotype responses to environmental variations (Saed-Moucheshi, Babaei & Ansarshourijeh, 2024; Kyratzis, Pallides & Katsiotis, 2022). Identifying genotypes with both high yield potential and stability across environments is a key objective in wheat breeding (Saed-Moucheshi, Pessarakli & Heidari, 2013). Our study showed that the first two components of the AMMI method were able to capture over 83% of the total variation of the grain yield data, consistent with previous research (Derbew et al., 2024; Abdelrahman et al., 2022; Saed-Moucheshi et al., 2017). High ratios of explaining the variance for these first two IPCA are crucial for the performance of the AMMI model (Farokhzadeh et al., 2022). According to the AMMI model, G122, G48, and G46 were the most stable genotypes across all environments. these genotypes were closest to the center of the IPCA plot (AMMI biplot) and had the lowest values of IPCA1 and IPCA2. In addition, AMMI model results regarding the grouping environments were the same as the heatmap-cluster result. Accordingly, Karaj and Nishapur were positioned in the same sector of the AMMI biplot, while they were significantly distanced from Zarghan and Kermanshah. In previous studies, AMMI has proven to be valuable in identifying superior genotypes under stress conditions, such as heat (Bishwas, Poudel & Regmi, 2021; Gupta et al., 2023) and drought (Barati et al., 2025) stresses. similarly, our study identified genotypes that performed well in drought-stressed environments and other normal conditions.

The GGE biplot analysis complemented the AMMI results by visually representing both genotype performance and stability. This model effectively identified mega-environments and the genotypes best suited to each separate environment, supporting previous research utilizing GGE biplots in wheat and other crops (Gupta et al., 2023; Nesa, Das & Sagor, 2024). GGE biplots offer a clearer understanding of genotype performance by focusing on genotype and G×E effects while minimizing the influence of environmental main effects (Omrani et al., 2024). Our analysis successfully categorized genotypes into broadly and specifically adapted groups, demonstrating the effectiveness of GGE in this context. In our study, the first PCA and the second PCA of the GGE biplot explained more than 75% of the variability among genotypes. In addition, the first PCA mainly contained the genotype effect, while the second PCA mostly contained the genotype-by-environment effect. Most studies with more than 15 genotypes showed significant PCA1 and PCA2 of GGE biplot with at least 60% explanation of the total variance (Aliakbari et al., 2013; Saed-Moucheshi et al., 2023; Rezaizad et al., 2025), which is consistent with our results. Additionally, the GGE biplot divided the result into seven sectors through which Kermanshah and Nishapur were placed in the same sector, separated from Karaj and Zarghan. However, Karaj and Nishapur were closest to the center, indicating low variability among the data from these two environments. Similar to our study, Omrani et al. (2022), Enyew et al. (2021), and Güngör et al. (2024) used a GGE biplot for clustering the tested environments by dividing the biplot into different sectors. Using these sectors enables us to identify the favorite genotypes for each environment, in addition to overall stable genotypes. Sectoring the GGE biplot showed that G106 and G76 were closer to the Kermanshah environment, and G155, G31, and G33 were closer to Zarghan compared to other genotypes. G125 and G76 were the closest genotypes to Karaj and Nishapur, respectively. All environments had positive values for the first biplot PCA, while both negative and positive values for the second biplot PCA. Therefore, genotypes with higher positive values of the first PCA and values close to zero for the second PCA can be identified as the favorite genotypes for all environments. Accordingly, G125, G39, G71, G161, G48, and G46 were the genotype favorites for all environments.

Integrating univariate and multivariate stability indices is essential for a comprehensive understanding of genotype stability and adaptability. While univariate indices provide basic stability information, multivariate approaches like AMMI and GGE biplot analysis capture intricate G×E interactions (Saed-Moucheshi, Babaei & Ansarshourijeh, 2024; Derbew et al., 2024; Saed-Moucheshi et al., 2019). Our study supports this integrated approach, as combining multiple methods allowed for a more comprehensive evaluation of genotype performance across environments. This comprehensive analysis facilitated the identification of promising genotypes exhibiting both high mean yield and stability, making them suitable candidates for future breeding efforts. These findings align with studies evaluating wheat genotypes under various stress conditions, where AMMI and GGE biplot analyses successfully identified superior lines (Saed-Moucheshi, Babaei & Ansarshourijeh, 2024; Bishwas, Poudel & Regmi, 2021). The selection of stable, high-yielding genotypes is crucial for developing resilient wheat cultivars capable of maintaining productivity in diverse and changing environments (Regmi et al., 2021; Biradar et al., 2024; Bishwas, Poudel & Regmi, 2021).

While the integrated analytical approach adopted in this study provides robust insights into genotype stability, some limitations and potential biases can be acknowledged. The augmented design, with unreplicated F_7_ lines, depends heavily on checks to control spatial variability. Although augmented designs are widely approved in early-generation trials, this approach inherently limits direct comparisons among lines and can introduce bias if checks perform inconsistently across blocks. Additionally, univariate indices such as the regression coefficient (b_*i*_) and deviation from regression (S^2^d_*i*_) favor certain types of stability (*e.g.*, average adaptability or minimal fluctuation), potentially down-weighting genotypes with high yield under specific favorable conditions. Similarly, AMMI and GGE biplots emphasize principal component variation but may oversimplify complex G×E patterns if lower-order interactions are biologically important, potentially biasing selection toward genotypes that perform well under dominant environmental contrasts while ignoring niche adaptation. This is the reason for applying the multivariate and univariate analytical methods for identifying the sable and high-performance genotypes in our study and providing an R-script for future similar studies.

From a physiological perspective, the identification of genotypes such as G48, G46, and G122 as stable high-yielders across contrasting environments likely reflects underlying mechanisms of drought resilience. For instance, consistent performance under terminal drought suggests traits such as enhanced osmotic adjustment, sustained chlorophyll content, and efficient root water uptake, attributes commonly associated with drought-tolerant wheat genotypes (Khandagale & Gawande, 2019). Moreover, genotypes derived from CIMMYT germplasm (*e.g.*, those from SAWSN/SAYWIT populations) showed superior performance under drought stress, supporting the value of broader genetic diversity and pre-breeding selection for adaptive physiological traits. Future research should complement stability analysis with measurements such as root architecture, leaf water potential, and antioxidant enzyme activities to validate these putative mechanisms (Gawande et al., 2017). This would strengthen the connection between statistical stability and underlying physiological resilience, providing more actionable insight for breeders.

The performance of wheat genotypes, particularly under drought stress, is significantly influenced by their root system architecture, where deep rooting allows access to moisture from lower soil layers, enhancing drought resilience (Strock & Schneider, 2022). Increased root density and well-distributed root systems facilitate efficient soil exploration for water and nutrients, which are crucial during water scarcity (Carbajal-Friedrich & Burgess, 2024). Additionally, genotypes that excel in osmotic adjustment by accumulating compatible solutes such as proline and glycine betaine can maintain turgor pressure under drought conditions, thereby sustaining cellular functions and enhancing yield potential (Ashraf & Foolad, 2007). Early maturity in certain genotypes enables them to escape terminal drought conditions, while the ability to synchronize developmental stages with favorable environmental conditions contributes to yield stability (Reynolds, 2001). High chlorophyll content and efficient photosynthesis are vital for maximizing grain yield, as these traits allow for continued photosynthesis during stress periods, leading to improved biomass accumulation (Morales et al., 2020). Furthermore, robust antioxidant mechanisms that scavenge reactive oxygen species help mitigate oxidative damage during drought stress, enhancing resilience and yield (Miller et al., 2010). Collectively, understanding these physiological traits can inform breeding programs aimed at developing wheat cultivars with enhanced resilience to variable environmental conditions.

## Conclusion

This study assessed the yield performance and stability of 165 F_7_ wheat genotypes and four check cultivars across four diverse environments in Iran, including a drought-stressed site in Nishapur. By incorporating both local and CIMMYT-derived genetic materials, the study leveraged broad genetic diversity to strengthen the evaluation of genotype adaptability. The use of univariate (*e.g.*, CV, SD, b_*i*_) and multivariate (AMMI, GGE biplot) stability indices revealed significant genotype-by-environment interactions. This combination also emphasized the necessity of employing complementary analytical methods to capture complex G×E dynamics. Genotypes G48, G46, and G122 were identified as both high-yielding and broadly adapted, showing consistent performance across environments and occupying central positions in AMMI and GGE biplots. While local genotypes performed well across environments, CIMMYT-derived lines demonstrated superior drought tolerance. This integrated analysis approach, supported by advanced visualization tools such as clustering and correlation heatmaps, proved effective in identifying resilient genotypes. The results offer valuable insights for breeding programs aiming to improve wheat yield stability under variable climatic conditions, particularly in regions prone to drought.

##  Supplemental Information

10.7717/peerj.20505/supp-1 Supplemental Information 1 Highlights

10.7717/peerj.20505/supp-2 Supplemental Information 2 Pedigree of the evaluated genotypes along with their codes

10.7717/peerj.20505/supp-3 Supplemental Information 3 Soil properties and total precipitation in all studied environments

10.7717/peerj.20505/supp-4 Supplemental Information 4 Univariate indices for each genotype across all environments

10.7717/peerj.20505/supp-5 Supplemental Information 5 Rank of each univariate stability index for each genotype across all environments

10.7717/peerj.20505/supp-6 Supplemental Information 6 The proportion of each component in genotype plus genotype by environment analysis

10.7717/peerj.20505/supp-7 Supplemental Information 7 Comparison plots according to the first and second components in GGE analysis by centering each environment in one plot

10.7717/peerj.20505/supp-8 Supplemental Information 8 Code for R programming Open in notepad.

10.7717/peerj.20505/supp-9 Supplemental Information 9 Complete R Code For Stbidx Library

10.7717/peerj.20505/supp-10 Supplemental Information 10 Dataset

## Additional Information and Declarations

### Data Availability

The following information was supplied regarding data availability: The data and code are available in the Supplemental File.
