# Supplementary material for: Evaluating multiple stability methods to screen bread wheat genotypes (F7 generation) under drought-stressed environments
Source: PeerJ. 2026 Feb 23;14:e20505. doi: 10.7717/peerj.20505 (PMC12939790; doi:10.7717/peerj.20505)
Supplement: Supplemental Information 1 [file peerj-14-20505-s001.docx]

Title: Evaluating Multiple Stability Methods to Screen Bread Wheat Genotypes (F7 Generation) Under Drought-Stressed Environments

Authors: Armin Saed-Moucheshi*, Shahryar Sesani, Farshad Bakhtiar, Davod Roodi, Shokoofeh Sarikhani Khorami

**Key Highlights**

- **Genetic Diversity:** The study evaluates 165 F7 wheat genotypes, integrating both **local (AREEO) and global (CIMMYT) wheat populations**, contributing to an enriched genetic pool.
- **Comprehensive Stability Assessment:** The study evaluates 165 F7 wheat genotypes and four check cultivars across four distinct environments, including a drought-stressed condition, using both univariate and multivariate stability indices.
- **Novel Analysis Methods:** This research **introduces advanced stability analyses**, including **heatmap clustering and correlation plots**, alongside **AMMI and GGE biplot models**, providing deeper insights into genotype stability.
- **New R-Based Computational Tool:** A **custom R script** for stability calculations is introduced, ensuring reproducibility and expanding accessibility for future research applications.
- **Identification of Stable Genotypes:** **G48, G46, and G122** emerged as **high-yielding, stable genotypes**, holding promise for wheat improvement programs under **drought stress conditions.**
- **Global Breeding Implications:** The study supports the integration of **CIMMYT-derived wheat genotypes** in breeding initiatives aimed at enhancing climate resilience and yield stability.

This work is **highly relevant to plant breeding, genotype-environment interaction studies, and wheat improvement programs worldwide.**
