# Supplementary material for: Evaluating multiple stability methods to screen bread wheat genotypes (F7 generation) under drought-stressed environments
Source: PeerJ. 2026 Feb 23;14:e20505. doi: 10.7717/peerj.20505 (PMC12939790; doi:10.7717/peerj.20505)
Supplement: Supplemental Information 2 [file peerj-14-20505-s002.docx]

| Supplementary Table 1. Pedigree of the evaluated genotypes along with their codes | | | |
| --- | --- | --- | --- |
| **Genotype ID** | **Pedigree original name** | **Pedigree** | **sources** |
| 1 | Bow"s"/Vee"s"//1-60-3/3/Cocoraque 75/4/Inia/5/Sirvan | IRW15-020331-3Kaj-0Kaj-0Kaj-2Kaj | F6- Karaj-2022 |
| 2 | Bow"s"/Vee"s"//1-60-3/3/Suweon 220/4/Chamran/5/Sirvan | IRW15-020333-1Kaj-0Kaj-0Kaj-1Kaj | F6- Karaj-2022 |
| 3 | Bow"s"/Vee"s"//1-60-3/3/Suweon 220/4/Chamran/5/Sirvan | IRW15-020333-1Kaj-0Kaj-0Kaj-2Kaj | F6- Karaj-2022 |
| 4 | Bow"s"/Vee"s"//1-60-3/3/Suweon 220/4/Chamran/5/Sirvan | IRW15-020333-6Kaj-0Kaj-0Kaj-1Kaj | F6- Karaj-2022 |
| 5 | PRL/2*PASTOR/4/CHOIX/STAR/3/HE1/3*CNO79//2*SERI/5/Baharan | IRW15-020335-2Kaj-0Kaj-0Kaj-2Kaj | F6- Karaj-2022 |
| 6 | PRL/2*PASTOR/4/CHOIX/STAR/3/HE1/3*CNO79//2*SERI/5/Baharan | IRW15-020335-5Kaj-0Kaj-0Kaj-2Kaj | F6- Karaj-2022 |
| 7 | W3918A/Jup//Shuha "s"/3/Shiraz/4/Rakhshan | IRW15-020341-1Kaj-0Kaj-0Kaj-2Kaj | F6- Karaj-2022 |
| 8 | W3918A/Jup//Shuha "s"/3/Shiraz/4/Rakhshan | IRW15-020341-1Kaj-0Kaj-0Kaj-3Kaj | F6- Karaj-2022 |
| 9 | W3918A/Jup//Shuha "s"/3/Shiraz/4/Rakhshan | IRW15-020341-3Kaj-0Kaj-0Kaj-1Kaj | F6- Karaj-2022 |
| 10 | MTRWA92.161/PRINIA/5/SERI*3//RL6010/4*YR/3/PASTOR/4/BAV92/6/Sirvan | IRW15-020351-2Kaj-0Kaj-0Kaj-3Kaj | F6- Karaj-2022 |
| 11 | CHEN/AEGILOPS SQUARROSA (TAUS)//BCN/3/BAV92/4/BERKUT/5/Sirvan | IRW15-020358-4Kaj-0Kaj-0Kaj-2Kaj | F6- Karaj-2022 |
| 12 | CHEN/AEGILOPS SQUARROSA (TAUS)//BCN/3/BAV92/4/BERKUT/5/Rakhshan | IRW15-020359-4Kaj-0Kaj-0Kaj-1Kaj | F6- Karaj-2022 |
| 13 | CHEN/AEGILOPS SQUARROSA (TAUS)//BCN/3/BAV92/4/BERKUT/5/Rakhshan | IRW15-020359-4Kaj-0Kaj-0Kaj-2Kaj | F6- Karaj-2022 |
| 14 | Nogal/Sirvan | IRW15-020380-3Kaj-0Kaj-0Kaj-1Kaj | F6- Karaj-2022 |
| 15 | Nogal/Sirvan | IRW15-020380-3Kaj-0Kaj-0Kaj-2Kaj | F6- Karaj-2022 |
| 16 | Tui//CMH 76-252/Pvn "s"/3/Flt/4/sirvan/5/Rakhshan | IRW15-020395-6Kaj-0Kaj-0Kaj-2Kaj | F6- Karaj-2022 |
| 17 | PRL/2*PASTOR/4/CHOIX/STAR/3/HE1/3*CNO79//2*SERI/5/Sirvan/6/Rakhshan | IRW15-020410-2Kaj-0Kaj-0Kaj-3Kaj | F6- Karaj-2022 |
| 18 | PRL/2*PASTOR/4/CHOIX/STAR/3/HE1/3*CNO79//2*SERI/5/Sirvan/6/Rakhshan | IRW15-020410-4Kaj-0Kaj-0Kaj-2Kaj | F6- Karaj-2022 |
| 19 | PRL/2*PASTOR/4/CHOIX/STAR/3/HE1/3*CNO79//2*SERI/5/Chamran2/6/Rakhshan | IRW15-020414-5Kaj-0Kaj-0Kaj-1Kaj | F6- Karaj-2022 |
| 20 | PRL/2*PASTOR/4/CHOIX/STAR/3/HE1/3*CNO79//2*SERI/5/Mehregan/6/Rakhshan | IRW15-020416-6Kaj-0Kaj-0Kaj-1Kaj | F6- Karaj-2022 |
| 21 | SERI.1B*2/3/KAUZ*2/BOW//KAUZ/4/VARIS/5/Pishgam/6/Sirvan | IRW15-020430-1Kaj-0Kaj-0Kaj-1Kaj | F6- Karaj-2022 |
| 22 | SERI.1B*2/3/KAUZ*2/BOW//KAUZ/4/VARIS/5/Pishgam/6/Sirvan | IRW15-020430-1Kaj-0Kaj-0Kaj-2Kaj | F6- Karaj-2022 |
| 23 | SERI.1B*2/3/KAUZ*2/BOW//KAUZ/4/PBW343*2/KHVAKI/5/Mehregan/6/Parsi | IRW15-020439-6Kaj-0Kaj-0Kaj-1Kaj | F6- Karaj-2022 |
| 24 | SERI.1B*2/3/KAUZ*2/BOW//KAUZ/4/PBW343*2/KHVAKI/5/Mehregan/6/Parsi | IRW15-020439-6Kaj-0Kaj-0Kaj-3Kaj | F6- Karaj-2022 |
| 25 | SERI.1B*2/3/KAUZ*2/BOW//KAUZ/4/PBW343*2/KHVAKI/5/Mehregan/6/Parsi | IRW15-020439-6Kaj-0Kaj-0Kaj-4Kaj | F6- Karaj-2022 |
| 26 | SOKOLL//W15.92/WBLL1/3/Sirvan/4/Rakhshan | IRW15-020449-3Kaj-0Kaj-0Kaj-2Kaj | F6- Karaj-2022 |
| 27 | SOKOLL//W15.92/WBLL1/3/Sirvan/4/Rakhshan | IRW15-020449-3Kaj-0Kaj-0Kaj-3Kaj | F6- Karaj-2022 |
| 28 | SOKOLL//W15.92/WBLL1/3/Sirvan/4/Rakhshan | IRW15-020449-4Kaj-0Kaj-0Kaj-1Kaj | F6- Karaj-2022 |
| 29 | SOKOLL//W15.92/WBLL1/3/Sirvan/4/Rakhshan | IRW15-020449-6Kaj-0Kaj-0Kaj-1Kaj | F6- Karaj-2022 |
| 30 | Celtic/Sirvan//Rakhshan | IRW15-020474-2Kaj-0Kaj-0Kaj-1Kaj | F6- Karaj-2022 |
| 31 | Heilo/Parsi//Sirvan | IRW15-020483-1Kaj-0Kaj-0Kaj-3Kaj | F6- Karaj-2022 |
| 32 | Shuha-8/Byt//Mehregan/3/Sirvan | IRW15-020388-0Ira-0Ard-0Ira-0A-1Kaj | F6- Karaj-2022 |
| 33 | BORL14//BECARD/QUAIU #1 | CMSS12Y00070S-099Y-099M-099NJ-099NJ-21Y-0WGY | 43th ESWYT |
| 34 | SHA7//PRL/VEE#6/3/FASAN/4/HAAS8446/2*FASAN/5/CBRD/KAUZ/6/MILAN/AMSEL/7/FRET2*2/KUKUNA/8/KINGBIRD #1/9/2*BORL14 | CMSS15Y01023T-099TOPM-099Y-099M-099NJ-099NJ-3Y-0WGY | 43th ESWYT |
| 35 | KACHU/SAUAL/5/KACHU/3/WHEAR//2*PRL/2*PASTOR/4/BOKOTA | CMSS15B00179S-099M-099NJ-099NJ-15Y-0WGY | 43th ESWYT |
| 36 | UP2338*2/VIVITSI/3/FRET2/TUKURU//FRET2/4/MISR 1/5/NADI | CMSS15B00243S-099M-099NJ-099NJ-18Y-0WGY | 43th ESWYT |
| 37 | CROSBILL #1/DANPHE/7/CNDO/R143//ENTE/MEXI_2/3/AEGILOPS SQUARROSA (TAUS)/4/WEAVER/5/2*KAUZ/6/PRL/2*PASTOR/8/NADI | CMSS15B00503S-099M-099NJ-099NJ-33Y-0WGY | 43th ESWYT |
| 38 | FRNCLN/3/ND643//2*PRL/2*PASTOR/4/FRANCOLIN #1*2/5/FRNCLN/NIINI #1//FRANCOLIN #1 | CMSS16Y00474T-099TOPM-099Y-099M-0SY-10M-0WGY | 43th ESWYT |
| 39 | PASTOR/KAUZ/6/CNDO/R143//ENTE/MEXI_2/3/AEGILOPS SQUARROSA (TAUS)/4/WEAVER/5/2*KAUZ/7/2*PRL/2*PASTOR//PBW343*2/KUKUNA/8/2*BORL14 | CMSS16Y00573T-099TOPM-099Y-099M-0SY-23M-0WGY | 43th ESWYT |
| 40 | PASTOR//HXL7573/2*BAU/3/SOKOLL/WBLL1/4/HUW234+LR34/PRINIA//PBW343*2/KUKUNA/3/ROLF07/5/WHEAR/SOKOLL/6/BORL14/7/KASUKO | CMSS16Y00659T-099TOPM-099Y-099M-0SY-34M-0WGY | 43th ESWYT |
| 41 | KACHU/SAUAL/3/TACUPETO F2001/BRAMBLING//KIRITATI*2/4/FRET2/TUKURU//FRET2/3/MUNAL #1 | CMSS16Y00714T-099TOPM-099Y-099M-0SY-37M-0WGY | 43th ESWYT |
| 42 | FRANCOLIN #1/3/PBW343*2/KUKUNA*2//YANAC/4/KINGBIRD #1//INQALAB 91*2/TUKURU*2/5/MUCUY | CMSS16Y00803T-099TOPM-099Y-099M-0SY-16M-0WGY | 43th ESWYT |
| 43 | SOKOLL/3/PASTOR//HXL7573/2*BAU*2/6/OASIS/5*BORL95/5/CNDO/R143//ENTE/MEXI75/3/AE.SQ/4/2*OCI*2/7/BORL14 | CMSS16Y00903T-099TOPM-099Y-099M-0SY-4M-0WGY | 43th ESWYT |
| 44 | YR57#5474-6/3*BORL14 | CMSS16B01875T-099Y-099M-099Y-32M-0WGY | 43th ESWYT |
| 45 | YR57#5474-6/3*BORL14 | CMSS16B01875T-099MABY-099M-099Y-1M-0WGY | 43th ESWYT |
| 46 | Pavon 76, 20´´ + 1R.1D5+10-2(1D)/3*MUCUY | CMSS16B01903T-099Y-099M-099Y-24M-0WGY | 43th ESWYT |
| 47 | CHAM-8/6/HUBARA-1/5/CHEN/AEGILOPS SQUARROSA(TAUS)//BCN/3/VEE#7/BOW/4/PASTOR | ISBW17-TR-0018-0TR-0TR-4MR-0MR | 23th ESBWYT |
| 48 | 02W50807-1/4/PFAU/SERI.1B//AMAD/3/WAXWING/5/BECARD//KIRITATI/2*TRCH/3/BECARD/4/NEJMAH-6/PAVON SR24+SR26+SR31 | ISBW17-TR-0066-0TR-0TR-7MR-0MR | 23th ESBWYT |
| 49 | RHINO 1A.1D5+10-4/TILHI//NEJMAH-14/4/SUDAN#3/SHUHA-6//FLAG-5/3/PFAU/MILAN | ISBW17-MR-677-0KUL-4MR-0MR | 23th ESBWYT |
| 50 | Misr-1/Angi-1 | ISBW17-MR-52-0KUL-3MR-0MR | 23th ESBWYT |
| 51 | QUAIU*2/KINDE/4/PFAU/MILAN/3/BABAX/LR42//BABAX | ISBW16MR-70-040MR-6MR-OMR | 23th ESBWYT |
| 52 | UTIQUE 96/FLAG-1//SR22CO1213/7/SERI.1B//KAUZ/HEVO/3/AMAD/4/PYN/BAU//MILAN/5/OPATA/RAYOM//KAUZ/6/SR50+SR45#1/8/TERBOL/9/DEBEIRA//MILAN/PASTOR/4/URES/BOW//OPATA/3/HT3306/HORK'S' | ISBW17S-TR-0125-0TR-0TR-4MR-0MR | 23th ESBWYT |
| 53 | COPIO*2/3/KINGBIRD#1//INQALAB 91*2TUKURU/4/BSKINA-8BONITO-36 | ISBWS18-TR-1228-0TR-0TR-6TR-0TR | 23th ESBWYT |
| 54 | DOY1/AE.SQUARROSA(1026)/5/SERI.1B*2/3/KAUZ*2/BOW//KAUZ/4/ANGI-26/6/PFAU/MILAN//ABIER-2/3/SHUHA-3//TURACO/CHIL | ISBW17-MR-187-0MR-5MR | 23th ESBWYT |
| 55 | KAUZ'S'/SERI//PFAU/MILAN/3/KFA/2*KACHU*2//WAXBI | ISBWS18-TR-0188-0TR-0TR-6TR-0TR | 23th ESBWYT |
| 56 | SAUAL/YANAC//SAUAL/3/BECARD/QUAIU#1/4/THELIN/WAXWING//ATTILA*2/PASTOR/3/INQALAB91*2/TUKURU9Y-0B | ISBWS18-TR-0531-0TR-0TR-1TR-0TR | 23th ESBWYT |
| 57 | KABILU #1 | CMSS10Y00374S-099Y-099M-1WGY-0B | 55th IBWSN |
| 58 | COPIO/MUCUY | CMSS15Y00360S-099Y-099M-099NJ-099NJ-16Y-0WGY | 55th IBWSN |
| 59 | FRNCLN/4/WHEAR/KUKUNA/3/C80.1/3*BATAVIA//2*WBLL1/5/2*SUP152*2/TECUE #1 | CMSS15Y00684T-099TOPM-099Y-099M-099NJ-099NJ-4Y-0WGY | 55th IBWSN |
| 60 | KACHU/SAUAL//CIRO16*2/4/WBLL1*2/BRAMBLING//TAM200/TUI/3/VILLA JUAREZ F2009 | CMSS15Y01083T-099TOPM-099Y-099M-099NJ-099NJ-24Y-0WGY | 55th IBWSN |
| 61 | BORL14/5/MUTUS/DANPHE #1/4/C80.1/3*BATAVIA//2*WBLL1/3/C80.1/3*QT4522//2*PASTOR | CMSS15B00150S-099M-099NJ-099NJ-41Y-0WGY | 55th IBWSN |
| 62 | CHIPAK/3/SWSR22T.B./2*BLOUK #1//WBLL1*2/KURUKU | CMSS15B00259S-099M-099NJ-099NJ-42Y-0WGY | 55th IBWSN |
| 63 | CHIPAK/4/KACHU/3/WHEAR//2*PRL/2*PASTOR | CMSS15B00261S-099M-099NJ-099NJ-15Y-0WGY | 55th IBWSN |
| 64 | KACHU//WBLL1*2/BRAMBLING/3/MUCUY | CMSS15B00266S-099M-099NJ-099NJ-23Y-0WGY | 55th IBWSN |
| 65 | PBW343*2/KUKUNA//PBW343*2/KUKUNA/3/WBLL1*2/SHAMA//KACHU/4/KASUKO | CMSS15B00619S-099M-099NJ-099NJ-42Y-0WGY | 55th IBWSN |
| 66 | SHA7//PRL/VEE#6/3/FASAN/4/HAAS8446/2*FASAN/5/CBRD/KAUZ/6/MILAN/AMSEL/7/FRET2*2/KUKUNA/8/TRCH/SRTU//KACHU/9/TRCH/HUIRIVIS #1/10/BORL14 | CMSS15B00920S-099M-099NJ-099NJ-18Y-0WGY | 55th IBWSN |
| 67 | KACHU/KIRITATI//BORL14/4/BECARD/AKURI*2/3/PBW343*2/KUKUNA*2//FRTL/PIFED | CMSS15B01367S-099M-099NJ-099NJ-17Y-0WGY | 55th IBWSN |
| 68 | MISR 1*2/3/KACHU//KIRITATI/2*TRCH | CMSS15B01901T-099TOPY-099M-099NJ-099NJ-17Y-0WGY | 55th IBWSN |
| 69 | MUTUS*2/KIRITATI//BORL14/3/MOKUE #1 | CMSS16Y00592T-099TOPM-099Y-099M-0SY-13M-0WGY | 55th IBWSN |
| 70 | SAUAL/WHEAR//SAUAL/3/PBW343*2/KUKUNA*2//FRTL/PIFED/4/BORL14/5/BECARD//ND643/2*WBLL1/4/ND643/2*WBLL1//ATTILA*2/PBW65/3/MUNAL | CMSS16Y00768T-099TOPM-099Y-099M-0SY-13M-0WGY | 55th IBWSN |
| 71 | FRANCOLIN #1/3/PBW343*2/KUKUNA*2//YANAC/4/KINGBIRD #1//INQALAB 91*2/TUKURU*2/5/BORL14 | CMSS16Y00793T-099TOPM-099Y-099M-0SY-22M-0WGY | 55th IBWSN |
| 72 | MUCUY/5/PBW65/2*PASTOR/3/KIRITATI//PBW65/2*SERI.1B/4/DANPHE #1/6/MOKUE #1 | CMSS16Y00362T-099TOPM-099Y-099M-099NJ-099NJ-8Y-0WGY | 17th STEMRRSN |
| 73 | FRANCOLIN #1/3/PBW343*2/KUKUNA*2//YANAC/4/KINGBIRD #1//INQALAB 91*2/TUKURU*2/5/BORL14 | CMSS16Y00793T-099TOPM-099Y-099M-099NJ-099NJ-25Y-0WGY | 17th STEMRRSN |
| 74 | FRANCOLIN #1/3/PBW343*2/KUKUNA*2//YANAC/4/KINGBIRD #1//INQALAB 91*2/TUKURU*2/5/MUNAL #1 | CMSS16Y00796T-099TOPM-099Y-099M-099NJ-099NJ-9Y-0WGY | 17th STEMRRSN |
| 75 | SUP152/FRNCLN//KASUKO | CMSS16B00100S-099M-0SY-099M-37Y-0WGY | 17th STEMRRSN |
| 76 | MUNAL*2/CHONTE//KASUKO | CMSS16B00134S-099M-0SY-099M-12Y-0WGY | 17th STEMRRSN |
| 77 | GLADIUS/3/2*KA/NAC//TRCH/4/KUTZ//KFA/2*KACHU | CMSS16B00147S-099M-099NJ-099NJ-4Y-0WGY | 17th STEMRRSN |
| 78 | KACHU*2/3/ND643//2*PRL/2*PASTOR/4/KASUKO | CMSS16B00170S-099M-0SY-099M-38Y-0WGY | 17th STEMRRSN |
| 79 | KACHU*2/3/ND643//2*PRL/2*PASTOR/4/MOKUE #1 | CMSS16B00173S-099M-0SY-099M-13Y-0WGY | 17th STEMRRSN |
| 80 | KACHU*2/3/ND643//2*PRL/2*PASTOR/4/MOKUE #1 | CMSS16B00173S-099M-099NJ-099NJ-1Y-0WGY | 17th STEMRRSN |
| 81 | KACHU/3/WHEAR//2*PRL/2*PASTOR/4/KASUKO | CMSS16B00230S-099M-0SY-099M-13Y-0WGY | 17th STEMRRSN |
| 82 | FRANCOLIN #1//WBLL1*2/KURUKU/3/WBLL1*2/BRAMBLING//CHYAK/4/SUP152//WBLL1*2/BRAMBLING*2/3/KSW/SAUAL//SAUAL | CMSS16B00859S-099M-0SY-099M-8Y-0WGY | 17th STEMRRSN |
| 83 | WAXWING/4/BL 1496/MILAN/3/CROC_1/AE.SQUARROSA (205)//KAUZ/5/FRNCLN/6/KINGBIRD #1//INQALAB 91*2/TUKURU/7/BECARD/QUAIU #1/8/2*KACHU//WBLL1*2/BRAMBLING*2/3/KACHU/KIRITATI | CMSS17Y00664T-099TOPM-099Y-099M-13Y-0WGY | 17th STEMRRSN |
| 84 | KACHU//WBLL1*2/BRAMBLING*2/6/ROLF07*2/5/REH/HARE//2*BCN/3/CROC_1/AE.SQUARROSA (213)//PGO/4/HUITES*2/7/KUTZ//KFA/2*KACHU | CMSS17Y00861T-099TOPM-099Y-099M-18Y-0WGY | 17th STEMRRSN |
| 85 | KACHU #1/3/T.DICOCCON PI94624/AE.SQUARROSA (409)//BCN/4/2*KACHU/5/MUTUS*2/TECUE #1/6/MUTUS*2/TECUE #1*2/7/NELOKI*2//KACHU/KIRITATI | CMSS17Y00997T-099TOPM-099Y-099M-11Y-0WGY | 17th STEMRRSN |
| 86 | KACHU #1/3/T.DICOCCON PI94624/AE.SQUARROSA (409)//BCN/4/2*KACHU/5/MUTUS*2/TECUE #1/6/MUTUS*2/TECUE #1*2/7/NELOKI*2//KACHU/KIRITATI | CMSS17Y00997T-099TOPM-099Y-099M-29Y-0WGY | 17th STEMRRSN |
| 87 | SAUAL*2/6/CNDO/R143//ENTE/MEXI_2/3/AEGILOPS SQUARROSA (TAUS)/4/WEAVER/5/2*PASTOR/7/PBW343*2/KUKUNA*2//FRTL/PIFED/8/BORL14/9/KASUKO | CMSS16Y00769T-099TOPM-099Y-099M-099NJ-099NJ-29Y-0RGY | 17th STEMRRSN |
| 88 | TACUPETO F2001/6/CNDO/R143//ENTE/MEXI_2/3/AEGILOPS SQUARROSA (TAUS)/4/WEAVER/5/PASTOR/7/ROLF07*2/8/SAUAL/YANAC//SAUAL/9/SUP152//WBLL1*2/BRAMBLING*2/3/KSW/SAUAL//SAUAL | CMSS16B00781S-099M-099NJ-099NJ-3Y-0RGY | 17th STEMRRSN |
| 89 | BORL14*2//BECARD/QUAIU #1/3/MOKUE #1 | CMSS16B00955S-099M-099NJ-099NJ-10Y-0RGY | 17th STEMRRSN |
| 90 | GRACK/CHYAK/6/ROLF07*2/5/FCT/3/GOV/AZ//MUS/4/DOVE/BUC/7/SUP152//WBLL1*2/BRAMBLING*2/3/KSW/SAUAL//SAUAL | CMSS16B01057S-099M-0SY-099M-11Y-0RGY | 17th STEMRRSN |
| 91 | BLOUK #1/MUNAL/3/WBLL1*2/SHAMA//BAJ #1/4/SUP152/BAJ #1/5/2*SUP152//WBLL1*2/BRAMBLING*2/3/KSW/SAUAL//SAUAL | CMSS17Y00907T-099TOPM-099Y-099M-2Y-0RGY | 17th STEMRRSN |
| 92 | CHEN/AEGILOPS SQUARROSA (TAUS)//BCN/3/BAV92/4/BERKUT/5/Parsi | 4-402-11 | PWSN Zaeghan |
| 93 | CHEN/AEGILOPS SQUARROSA (TAUS)//BCN/3/BAV92/4/BERKUT/5/Parsi | 4-402-12 | PWSN Zaeghan |
| 94 | CHEN/AEGILOPS SQUARROSA (TAUS)//BCN/3/BAV92/4/BERKUT/5/Parsi | 4-402-13 | PWSN Zaeghan |
| 95 | CHEN/AEGILOPS SQUARROSA (TAUS)//BCN/3/BAV92/4/BERKUT/5/Parsi | 4-402-14 | PWSN Zaeghan |
| 96 | CHEN/AEGILOPS SQUARROSA (TAUS)//BCN/3/BAV92/4/BERKUT/5/Sirvan | 4-402-17 | PWSN Zaeghan |
| 97 | Nogal/Sivand | 4-402-25 | PWSN Zaeghan |
| 98 | Tui//CMH 76-252/Pvn "s"/3/Flt/4/Parsi/5/Rakhshan | 4-402-32 | PWSN Zaeghan |
| 99 | PRL/2*PASTOR/4/CHOIX/STAR/3/HE1/3*CNO79//2*SERI/5/Pishgam/6/Parsi | 4-402-42 | PWSN Zaeghan |
| 100 | PRL/2*PASTOR/4/CHOIX/STAR/3/HE1/3*CNO79//2*SERI/5/Chamran2/6/Parsi | 4-402-45 | PWSN Zaeghan |
| 101 | WHEAR/KUKUNA/3/C80.1/3*BATAVIA//2*WBLL1/4/Parsi/5/Rakhshan | 4-402-53 | PWSN Zaeghan |
| 102 | WHEAR/KUKUNA/3/C80.1/3*BATAVIA//2*WBLL1/4/Parsi/5/Rakhshan | 4-402-54 | PWSN Zaeghan |
| 103 | SERI.1B*2/3/KAUZ*2/BOW//KAUZ/4/VARIS/5/Sirvan/6/Rakhshan | 4-402-62 | PWSN Zaeghan |
| 104 | SERI.1B*2/3/KAUZ*2/BOW//KAUZ/4/PBW343*2/KHVAKI/5/Chamran2/6/Rakhshan | 4-402-70 | PWSN Zaeghan |
| 105 | ATTILA*2/PBW65//BERKUT/3/Pishgam/4/Sirvan | 4-402-81 | PWSN Zaeghan |
| 106 | SOKOLL//W15.92/WBLL1/3/Sirvan/4/Rakhshan | 4-402-91 | PWSN Zaeghan |
| 107 | PASTOR//SITE/MO/3/CHEN/AEGILOPS SQUARROSA (TAUS)//BCN/4/WBLL1/5/Pishgam/6/Sirvan | 4-402-101 | PWSN Zaeghan |
| 108 | KS82W418/SPN/3/CHEN/AE.SQ//2*OPATA/4/FRET2/5/Parsi/6/Sirvan | 4-402-103 | PWSN Zaeghan |
| 109 | Heilo/Pishtaz//Parsi | 4-402-122 | PWSN Zaeghan |
| 110 | Heilo/Chamran2//Parsi | 4-402-124 | PWSN Zaeghan |
| 111 | WEAVER/TSC//WEAVER/3/WEAVER/4/2*WAXWING/5/Baharan | 4-402-145 | PWSN Zaeghan |
| 112 | PRL/2*PASTOR//Baharan | 4-402-151 | PWSN Zaeghan |
| 113 | QAFZAH-14/ASFOOR-1//Baharan | 4-402-159 | PWSN Zaeghan |
| 114 | PASTOR/KAUZ/6/CNDO/R143//ENTE/MEXI_2/3/AEGILOPS SQUARROSA (TAUS)/4/WEAVER/5/2*KAUZ/7/SOKOLL/WBLL1 | 4-402-172 | PWSN Zaeghan |
| 115 | HUBARA-8/3/MON'S'/ALD'S'//BOW'S'/4/SOKOLL/WBLL1 | 4-402-173 | PWSN Zaeghan |
| 116 | QAFZAH-33/FLORKWA-2//SOKOLL/WBLL1 | 4-402-175 | PWSN Zaeghan |
| 117 | HAALA-1//SOKOLL/WBLL1 | 4-402-180 | PWSN Zaeghan |
| 118 | HAALA-1//SOKOLL/WBLL1 | 4-402-181 | PWSN Zaeghan |
| 119 | HAALA-37//SOKOLL/WBLL1 | 4-402-182 | PWSN Zaeghan |
| 120 | BABAGA-3//SOKOLL/WBLL1 | 4-402-194 | PWSN Zaeghan |
| 121 | REBWAH-12/ZEMAMRA-8//Pishtaz | 4-402-196 | PWSN Zaeghan |
| 122 | REBWAH-12/ZEMAMRA-8//Pishtaz | 4-402-199 | PWSN Zaeghan |
| 123 | SETTAT-69/Pishtaz | 4-402-201 | PWSN Zaeghan |
| 124 | SETTAT-76/Pishtaz | 4-402-203 | PWSN Zaeghan |
| 125 | SOKOLL/3/PASTOR//HXL7573/2*BAU*2/6/OASIS/5*BORL95/5/CNDO/R143//ENTE/MEXI75/3/AE.SQ/4/2*OCI | CMSA10M00159T-050Y-099ZTM-099NJ-099NJ-5WGY-0B | 40th SAWSN |
| 126 | KACHU #1//WBLL1*2/KUKUNA/3/BRBT1*2/KIRITATI/6/ROLF07*2/5/REH/HARE//2*BCN/3/CROC_1/AE.SQUARROSA (213)//PGO/4/HUITES/7/BORL14 | CMSS12B01029T-099TOPY-099M-099NJ-099NJ-20Y-0WGY | 40th SAWSN |
| 127 | KABILU #1*2/TAITA | CMSS15Y01080T-099TOPM-099Y-099M-099NJ-099NJ-26Y-0WGY | 40th SAWSN |
| 128 | CNDO/R143//ENTE/MEXI_2/3/AEGILOPS SQUARROSA (TAUS)/4/WEAVER/5/2*JANZ/6/SKAUZ/BAV92/7/TRCH/3/ROLF07/YANAC//TACUPETO F2001/BRAMBLING/4/PRL/2*PASTOR | CMSS15B00758S-099M-099NJ-099NJ-22Y-0WGY | 40th SAWSN |
| 129 | SOKOLL/3/PASTOR//HXL7573/2*BAU/4/SHAMA//PARUS/PASTOR/5/BORL14 | CMSS15B01036S-099M-099NJ-099NJ-4Y-0WGY | 40th SAWSN |
| 130 | WBLL1*2/KUKUNA*2//WHEAR/8/2*TACUPETO F2001/6/CNDO/R143//ENTE/MEXI_2/3/AEGILOPS SQUARROSA (TAUS)/4/WEAVER/5/PASTOR/7/ROLF07/9/KFA/2*KACHU/3/PBW343*2/KUKUNA*2//FRTL/PIFED/4/KFA/2*KACHU | CMSS15B01305S-099M-099NJ-099NJ-7Y-0WGY | 40th SAWSN |
| 131 | BECARD/AKURI*2/4/MUU #1//PBW343*2/KUKUNA/3/MUU/5/KUTZ//KFA/2*KACHU | CMSS15B01320S-099M-099NJ-099NJ-42Y-0WGY | 40th SAWSN |
| 132 | NADI//KACHU/KIRITATI/3/NADI#2 | CMSS15B01958T-099TOPY-099M-099NJ-099NJ-17Y-0WGY | 40th SAWSN |
| 133 | MUNAL #1/SUJATA//CHIPAK | CMSS15B02016T-099TOPY-099M-099NJ-099NJ-15Y-0WGY | 40th SAWSN |
| 134 | ATTILA*2/PBW65/5/PRL/2*PASTOR/4/CHOIX/STAR/3/HE1/3*CNO79//2*SERI/6/PFUNYE #1/7/BORL14/8/MELON//FILIN/MILAN/3/FILIN/4/TRCH/SRTU//KACHU | CMSS16Y00562T-099TOPM-099Y-099M-0SY-13M-0WGY | 40th SAWSN |
| 135 | MUTUS*2/KIRITATI//BORL14/3/MOKUE #1 | CMSS16Y00592T-099TOPM-099Y-099M-0SY-16M-0WGY | 40th SAWSN |
| 136 | MUNAL #1/CIRO16*2//KACHU/KIRITATI | CMSS16Y00694T-099TOPM-099Y-099M-0SY-16M-0WGY | 40th SAWSN |
| 137 | SAUAL*2/6/CNDO/R143//ENTE/MEXI_2/3/AEGILOPS SQUARROSA (TAUS)/4/WEAVER/5/2*PASTOR/7/PBW343*2/KUKUNA*2//FRTL/PIFED/8/BORL14/9/KASUKO | CMSS16Y00769T-099TOPM-099Y-099M-0SY-17M-0WGY | 40th SAWSN |
| 138 | KACHU/SAUAL*2/4/ATTILA*2/PBW65//PIHA/3/ATTILA/2*PASTOR/5/SOKOLL/3/PASTOR//HXL7573/2*BAU/4/SOKOLL//PBW343*2/KUKUNA/3/NAVJ07/8/OASIS/5*BORL95/5/CNDO/R143//ENTE/MEXI75/3/AE.SQ/4/2*OCI/6/SOKOLL//SUNCO/2*PASTOR/7/SOKOLL//SUNCO/2*PASTOR | CMSS16Y00862T-099TOPM-099Y-099M-0SY-33M-0WGY | 40th SAWSN |
| 139 | WAXWING/KIRITATI//FISCAL/3/HUW234+LR34/PRINIA//UP2338*2/VIVITSI/4/HUW234+LR34/PRINIA*2//YANAC*2/5/FRANCOLIN #1/3/PBW343*2/KUKUNA*2//YANAC/4/KINGBIRD #1//INQALAB 91*2/TUKURU | CMSS16Y00867T-099TOPM-099Y-099M-0SY-1M-0WGY | 40th SAWSN |
| 140 | SOKOLL/3/PASTOR//HXL7573/2*BAU/4/SHAMA//PARUS/PASTOR/5/BORL14/7/SOKOLL/3/PASTOR//HXL7573/2*BAU*2/6/OASIS/5*BORL95/5/CNDO/R143//ENTE/MEXI75/3/AE.SQ/4/2*OCI | CMSS16Y00887T-099TOPM-099Y-099M-0SY-24M-0WGY | 40th SAWSN |
| 141 | OASIS/5*BORL95/5/CNDO/R143//ENTE/MEXI75/3/AE.SQ/4/2*OCI/6/SOKOLL//SUNCO/2*PASTOR/7/SOKOLL//SUNCO/2*PASTOR*2/8/CROSBILL #1/DANPHE/7/CNDO/R143//ENTE/MEXI_2/3/AEGILOPS SQUARROSA (TAUS)/4/WEAVER/5/2*KAUZ/6/PRL/2*PASTOR | CMSS16Y00897T-099TOPM-099Y-099M-0SY-9M-0WGY | 40th SAWSN |
| 142 | SOKOLL/3/PASTOR//HXL7573/2*BAU*2/6/OASIS/5*BORL95/5/CNDO/R143//ENTE/MEXI75/3/AE.SQ/4/2*OCI*2/7/FRANCOLIN #1/3/PBW343*2/KUKUNA*2//YANAC/4/KINGBIRD #1//INQALAB 91*2/TUKURU | CMSS16Y00901T-099TOPM-099Y-099M-0SY-1M-0WGY | 40th SAWSN |
| 143 | SOKOLL/3/PASTOR//HXL7573/2*BAU*2/6/OASIS/5*BORL95/5/CNDO/R143//ENTE/MEXI75/3/AE.SQ/4/2*OCI*2/7/BORL14 | CMSS16Y00903T-099TOPM-099Y-099M-0SY-16M-0WGY | 40th SAWSN |
| 144 | CROC_1/AE.SQUARROSA (224)//OPATA/3/PASTOR/4/2*SOKOLL/3/PASTOR//HXL7573/2*BAU/5/MUTUS*2/TECUE #1/6/MOKUE #1 | CMSS16Y00911T-099TOPM-099Y-099M-0SY-33M-0WGY | 40th SAWSN |
| 145 | YR57#5474-6/3*BORL14 | CMSS16B01875T-099MABY-099M-099Y-1M-0WGY | 40th SAWSN |
| 146 | SR47/5/3*SHORTENED SR26 TRANSLOCATION/4/3*CHIBIA//PRLII/CM65531/3/MISR 2 | CMSS16B01888T-099Y-099M-099Y-11M-0WGY | 40th SAWSN |
| 147 | CHUAN NONG 19/3*MISR 1 | CMSS16B01892T-099Y-099M-099Y-22M-0WGY | 40th SAWSN |
| 148 | Pavon 76, 20´´ + 1R.1D5+10-2(1D)/3*MUCUY | CMSS16B01903T-099Y-099M-099Y-1M-0WGY | 40th SAWSN |
| 149 | Pavon 76, 20´´ + 1RSe.1AL/BORL14/3/2*BORL14//KFA/2*KACHU | CMSS16B01905T-099Y-099M-099Y-6M-0WGY | 40th SAWSN |
| 150 | SOKOLL/3/PASTOR//HXL7573/2*BAU*2/6/OASIS/5*BORL95/5/CNDO/R143//ENTE/MEXI75/3/AE.SQ/4/2*OCI | CMSA10M00159T-050Y-099ZTM-099NJ-099NJ-5WGY-0B | 30th SAWYT |
| 151 | SUP152/BAJ #1/5/PBW65/2*PASTOR/3/KIRITATI//PBW65/2*SERI.1B/4/DANPHE #1 | CMSS15Y00409S-099Y-099M-099NJ-099NJ-9Y-0WGY | 30th SAWYT |
| 152 | BAJ #1*2/WHEAR*2/3/PRL/2*PASTOR*2//FH6-1-7 | CMSS15Y00623T-099TOPM-099Y-099M-099NJ-099NJ-13Y-0WGY | 30th SAWYT |
| 153 | MUTUS*2/TECUE #1*2//KFA/2*KACHU | CMSS15Y00674T-099TOPM-099Y-099M-099NJ-099NJ-30Y-0WGY | 30th SAWYT |
| 154 | KABILU #1*2/TAITA | CMSS15Y01080T-099TOPM-099Y-099M-099NJ-099NJ-26Y-0WGY | 30th SAWYT |
| 155 | BORL14/5/MUTUS/DANPHE #1/4/C80.1/3*BATAVIA//2*WBLL1/3/C80.1/3*QT4522//2*PASTOR | CMSS15B00150S-099M-099NJ-099NJ-21Y-0WGY | 30th SAWYT |
| 156 | CHIPAK/3/SWSR22T.B./2*BLOUK #1//WBLL1*2/KURUKU | CMSS15B00259S-099M-099NJ-099NJ-42Y-0WGY | 30th SAWYT |
| 157 | KACHU/BECARD//WBLL1*2/BRAMBLING/3/KACHU//KIRITATI/2*TRCH | CMSS15B00325S-099M-099NJ-099NJ-14Y-0WGY | 30th SAWYT |
| 158 | KACHU//KIRITATI/2*TRCH/3/CHIPAK | CMSS15B00350S-099M-099NJ-099NJ-24Y-0WGY | 30th SAWYT |
| 159 | TRCH/3/ROLF07/YANAC//TACUPETO F2001/BRAMBLING/4/PRL/2*PASTOR/5/BORL14 | CMSS15B00822S-099M-099NJ-099NJ-29Y-0WGY | 30th SAWYT |
| 160 | BECARD/AKURI*2/4/MUU #1//PBW343*2/KUKUNA/3/MUU/5/KUTZ//KFA/2*KACHU | CMSS15B01320S-099M-099NJ-099NJ-42Y-0WGY | 30th SAWYT |
| 161 | WBLL1*2/SHAMA//BAJ #1/3/BORL14/4/KASUKO | CMSS15B01359S-099M-099NJ-099NJ-10Y-0WGY | 30th SAWYT |
| 162 | SOKOLL/3/PASTOR//HXL7573/2*BAU/4/SHAMA//PARUS/PASTOR/5/BORL14/7/SOKOLL/3/PASTOR//HXL7573/2*BAU*2/6/OASIS/5*BORL95/5/CNDO/R143//ENTE/MEXI75/3/AE.SQ/4/2*OCI | CMSS16Y00887T-099TOPM-099Y-099M-0SY-24M-0WGY | 30th SAWYT |
| 163 | SOKOLL/3/PASTOR//HXL7573/2*BAU*2/6/OASIS/5*BORL95/5/CNDO/R143//ENTE/MEXI75/3/AE.SQ/4/2*OCI*2/7/BORL14 | CMSS16Y00903T-099TOPM-099Y-099M-0SY-16M-0WGY | 30th SAWYT |
| 164 | YR57#5474-6/3*BORL14 | CMSS16B01875T-099Y-099M-099Y-36M-0WGY | 30th SAWYT |
| 165 | SWSR22T.B./5/KAUZ//ALTAR 84/AOS/3/KAUZ/4/SW94.15464/6/2*PRL/2*PASTOR/7/WA8124/8/BAJ #1/CIRO16 | CMSS16Y01136T-099TOPM-099Y-099M-099Y-45M-0WGY | 30th SAWYT |
