# Supplementary material for: Evaluating multiple stability methods to screen bread wheat genotypes (F7 generation) under drought-stressed environments
Source: PeerJ. 2026 Feb 23;14:e20505. doi: 10.7717/peerj.20505 (PMC12939790; doi:10.7717/peerj.20505)
Supplement: Supplemental Information 3 [file peerj-14-20505-s003.docx]

| Supplementary Table 2. Soil properties and total precipitation in all studied environments. | | | | |
| --- | --- | --- | --- | --- |
| **Soil Property** | **Kermanshah** | **Nishapur** | Karaj | Zarghan |
| Soil Type | Sandy-Clay | Sandy-Clay | Sandy-Clay | Sandy-Clay |
| pH | 6.7 | 6.1 | 6.4 | 5.8 |
| Organic Matter (%) | 0.90 | 0.75 | 0.80 | 0.60 |
| Bulk Density (g/cm³) | 1.45 | 1.35 | 1.55 | 1.10 |
| Cation Exchange Capacity (cmol/kg) | 14 | 13 | 15 | 16 |
| Precipitation (mm) | 340 | 195 | 340 | 245 |
