# Supplementary material for: Evaluating multiple stability methods to screen bread wheat genotypes (F7 generation) under drought-stressed environments
Source: PeerJ. 2026 Feb 23;14:e20505. doi: 10.7717/peerj.20505 (PMC12939790; doi:10.7717/peerj.20505)
Supplement: Supplemental Information 4 [file peerj-14-20505-s004.docx]

| Univariate indices for each genotype across all environments. | | | | | | | | |
| --- | --- | --- | --- | --- | --- | --- | --- | --- |
| Genotype | Yi | S2 | CV | W2 | sig2 | b | sd | R2 |
| 1 | 5.685 | 1.504 | 21.57 | 3.174 | 0.02653 | 0.7918 | 3.031 | 0.5076 |
| 2 | 6.914 | 1.952 | 20.21 | 2.402 | 0.02004 | 1.0547 | 4.599 | 0.6942 |
| 3 | 6.929 | 0.923 | 13.87 | 0.302 | 0.00248 | 0.8479 | 2.543 | 0.9487 |
| 4 | 6.947 | 1.966 | 20.19 | 5.079 | 0.04249 | 0.786 | 3.441 | 0.3826 |
| 5 | 6.89 | 0.635 | 11.56 | 0.916 | 0.00765 | 0.6665 | 1.657 | 0.8525 |
| 6 | 7.456 | 0.494 | 9.43 | 1.293 | 0.01082 | 0.57 | 1.251 | 0.8013 |
| 7 | 6.941 | 1.104 | 15.14 | 0.689 | 0.00572 | 0.8824 | 2.894 | 0.8592 |
| 8 | 6.428 | 1.666 | 20.08 | 1.772 | 0.01477 | 1.0021 | 4.037 | 0.7341 |
| 9 | 7.16 | 2.143 | 20.44 | 1.709 | 0.01422 | 1.2043 | 5.503 | 0.8243 |
| 10 | 7.674 | 1.973 | 18.3 | 1.345 | 0.01118 | 1.172 | 5.139 | 0.8479 |
| 11 | 7.271 | 1.868 | 18.8 | 1.664 | 0.01386 | 1.0963 | 4.678 | 0.7834 |
| 12 | 7.234 | 1.606 | 17.52 | 8.057 | 0.06748 | 0.3323 | 1.315 | 0.0838 |
| 13 | 6.928 | 0.806 | 12.96 | 2.632 | 0.02203 | 0.5609 | 1.572 | 0.4752 |
| 14 | 6.109 | 0.336 | 9.49 | 2.558 | 0.02143 | 0.3755 | 0.68 | 0.5109 |
| 15 | 6.409 | 0.801 | 13.97 | 2.924 | 0.02448 | 0.529 | 1.478 | 0.4252 |
| 16 | 7.39 | 1.556 | 16.88 | 0.428 | 0.0035 | 1.0948 | 4.263 | 0.9383 |
| 17 | 6.688 | 1.808 | 20.1 | 3.163 | 0.02643 | 0.9176 | 3.852 | 0.5672 |
| 18 | 7.18 | 0.651 | 11.24 | 2.281 | 0.0191 | 0.5331 | 1.342 | 0.5317 |
| 19 | 6.32 | 1.78 | 21.11 | 9.173 | 0.07683 | 0.2891 | 1.204 | 0.0572 |
| Danesh | 7.294 | 0.716 | 11.6 | 2.608 | 0.02183 | 0.5262 | 1.389 | 0.4711 |
| 21 | 6.427 | 0.575 | 11.8 | 1.422 | 0.01189 | 0.5902 | 1.397 | 0.7375 |
| 22 | 6.412 | 0.555 | 11.62 | 3.408 | 0.02855 | 0.378 | 0.879 | 0.3136 |
| 23 | 6.542 | 1.049 | 15.66 | 1.975 | 0.01651 | 0.7281 | 2.328 | 0.6153 |
| 24 | 6.568 | 2.031 | 21.7 | 2.025 | 0.01688 | 1.1258 | 5.008 | 0.7602 |
| 25 | 7.037 | 0.741 | 12.23 | 0.255 | 0.0021 | 0.7778 | 2.089 | 0.9949 |
| 26 | 7.03 | 1.239 | 15.84 | 0.193 | 0.00156 | 0.9889 | 3.437 | 0.9611 |
| 27 | 7.412 | 2 | 19.08 | 0.516 | 0.00422 | 1.268 | 5.597 | 0.9792 |
| 28 | 6.067 | 2.086 | 23.8 | 0.525 | 0.0043 | 1.3025 | 5.872 | 0.9905 |
| 29 | 6.312 | 2.738 | 26.21 | 1.985 | 0.0165 | 1.4202 | 7.335 | 0.8973 |
| 30 | 6.192 | 2.394 | 24.99 | 1.902 | 0.01582 | 1.2875 | 6.218 | 0.8435 |
| 31 | 7.068 | 3.158 | 25.14 | 6.408 | 0.05357 | 1.1387 | 6.316 | 0.5001 |
| 32 | 6.603 | 2.305 | 23 | 1.431 | 0.01188 | 1.2995 | 6.159 | 0.8922 |
| 33 | 7.019 | 2.445 | 22.28 | 6.732 | 0.05632 | 0.8128 | 3.967 | 0.3291 |
| 34 | 6.156 | 2.23 | 24.25 | 0.681 | 0.0056 | 1.3454 | 6.271 | 0.9888 |
| 35 | 6.189 | 1.925 | 22.42 | 1.092 | 0.00906 | 1.1783 | 5.103 | 0.8783 |
| 36 | 6.228 | 1.593 | 20.26 | 2.32 | 0.01937 | 0.9158 | 3.608 | 0.6413 |
| 37 | 6.854 | 1.992 | 20.59 | 1.164 | 0.00966 | 1.1984 | 5.28 | 0.878 |
| 38 | 5.838 | 0.991 | 17.05 | 0.849 | 0.00707 | 0.8198 | 2.548 | 0.8258 |
| 39 | 5.675 | 2.934 | 30.18 | 3.174 | 0.02647 | 1.3789 | 7.373 | 0.7891 |
| Amin | 8.094 | 1.429 | 14.77 | 0.534 | 0.0044 | 1.032 | 3.851 | 0.9074 |
| 41 | 6.73 | 3.763 | 28.82 | 5.921 | 0.04945 | 1.4372 | 8.703 | 0.6685 |
| 42 | 7.155 | 2.118 | 20.34 | 1.642 | 0.01366 | 1.2008 | 5.455 | 0.8294 |
| 43 | 6.923 | 1.699 | 18.83 | 2.071 | 0.01728 | 0.9849 | 4.007 | 0.6954 |
| 44 | 6.026 | 1.618 | 21.11 | 2.267 | 0.01892 | 0.9315 | 3.698 | 0.6533 |
| 45 | 6.704 | 1.191 | 16.28 | 1.112 | 0.00926 | 0.8748 | 2.98 | 0.7826 |
| 46 | 6.059 | 0.526 | 11.97 | 1.446 | 0.0121 | 0.5675 | 1.285 | 0.7459 |
| 47 | 7.282 | 1.101 | 14.41 | 1.609 | 0.01343 | 0.7871 | 2.578 | 0.685 |
| 48 | 6.112 | 1.229 | 18.14 | 0.026 | 0.00015 | 1.002 | 3.468 | 0.9947 |
| 49 | 6.698 | 0.911 | 14.25 | 1.256 | 0.01049 | 0.745 | 2.219 | 0.7422 |
| 50 | 6.694 | 1.347 | 17.33 | 0.16 | 0.00127 | 1.0364 | 3.754 | 0.9716 |
| 51 | 6.843 | 0.959 | 14.31 | 1.266 | 0.01056 | 0.7638 | 2.335 | 0.7409 |
| 52 | 7.062 | 0.971 | 13.95 | 1.479 | 0.01236 | 0.7466 | 2.296 | 0.6995 |
| 53 | 7.206 | 0.805 | 12.45 | 0.661 | 0.0055 | 0.7627 | 2.136 | 0.8801 |
| 54 | 7.428 | 2.727 | 22.23 | 4.423 | 0.03695 | 1.1657 | 6.009 | 0.6068 |
| 55 | 7.792 | 0.77 | 11.26 | 2.612 | 0.02187 | 0.5478 | 1.5 | 0.4749 |
| 56 | 7.377 | 0.946 | 13.18 | 2.045 | 0.0171 | 0.6785 | 2.06 | 0.5927 |
| 57 | 7.481 | 2.308 | 20.3 | 2.644 | 0.02205 | 1.176 | 5.576 | 0.73 |
| 58 | 7.653 | 1.866 | 17.85 | 1.619 | 0.01348 | 1.0999 | 4.69 | 0.7896 |
| 59 | 7.694 | 2.045 | 18.59 | 1.669 | 0.01389 | 1.1684 | 5.216 | 0.8129 |
| Farin | 7.155 | 3.807 | 27.27 | 4.175 | 0.03481 | 1.6342 | 9.953 | 0.8545 |
| 61 | 7.512 | 2.113 | 19.35 | 1.716 | 0.01428 | 1.1914 | 5.406 | 0.8181 |
| 62 | 7.846 | 2.095 | 18.45 | 2.637 | 0.022 | 1.0893 | 4.921 | 0.6899 |
| 63 | 6.849 | 1.168 | 15.78 | 1.925 | 0.01608 | 0.7819 | 2.638 | 0.6375 |
| 64 | 7.132 | 1.891 | 19.28 | 3.317 | 0.02772 | 0.9357 | 4.016 | 0.5641 |
| 65 | 6.967 | 2.149 | 21.04 | 1.06 | 0.00878 | 1.2732 | 5.826 | 0.9189 |
| 66 | 6.644 | 1.404 | 17.83 | 0.717 | 0.00594 | 1.0028 | 3.709 | 0.8724 |
| 67 | 7.213 | 1.098 | 14.53 | 0.546 | 0.00452 | 0.8947 | 2.926 | 0.8879 |
| 68 | 6.916 | 1.903 | 19.95 | 1.653 | 0.01376 | 1.1118 | 4.788 | 0.7909 |
| 69 | 7.355 | 1.205 | 14.93 | 0.71 | 0.00589 | 0.922 | 3.16 | 0.859 |
| 70 | 7.403 | 1.362 | 15.76 | 0.553 | 0.00457 | 1.0022 | 3.65 | 0.8985 |
| 71 | 8.138 | 2.011 | 17.42 | 0.899 | 0.00743 | 1.2332 | 5.459 | 0.9212 |
| 72 | 5.473 | 2.069 | 26.28 | 4.384 | 0.03665 | 0.8996 | 4.039 | 0.4763 |
| 73 | 7.564 | 3.34 | 24.16 | 3.585 | 0.02989 | 1.5032 | 8.575 | 0.824 |
| 74 | 7.646 | 1.733 | 17.22 | 1.248 | 0.01037 | 1.0835 | 4.453 | 0.8249 |
| 75 | 7.28 | 2.273 | 20.71 | 2.119 | 0.01765 | 1.2155 | 5.72 | 0.7918 |
| 76 | 7.506 | 3.96 | 26.51 | 5.225 | 0.04361 | 1.5892 | 9.871 | 0.7769 |
| 77 | 7.656 | 1.131 | 13.89 | 2.074 | 0.01733 | 0.7516 | 2.495 | 0.6082 |
| 78 | 6.36 | 2.368 | 24.2 | 5.366 | 0.04487 | 0.9216 | 4.427 | 0.4368 |
| 79 | 7.135 | 2.121 | 20.41 | 0.949 | 0.00785 | 1.2731 | 5.787 | 0.931 |
| Torabi | 7.199 | 1.442 | 16.68 | 0.648 | 0.00536 | 1.0254 | 3.843 | 0.8881 |
| 81 | 6.745 | 2.17 | 21.84 | 1.717 | 0.01428 | 1.2147 | 5.586 | 0.8281 |
| 82 | 7.256 | 1.98 | 19.39 | 1.091 | 0.00904 | 1.2008 | 5.274 | 0.8871 |
| 83 | 7.342 | 1.202 | 14.93 | 0.162 | 0.00129 | 0.9768 | 3.342 | 0.9669 |
| 84 | 7.182 | 1.018 | 14.05 | 0.908 | 0.00756 | 0.8249 | 2.598 | 0.8139 |
| 85 | 7.872 | 3.466 | 23.65 | 3.051 | 0.02541 | 1.6096 | 9.354 | 0.9105 |
| 86 | 6.841 | 1.17 | 15.81 | 1.059 | 0.00882 | 0.8718 | 2.944 | 0.791 |
| 87 | 6.598 | 0.698 | 12.66 | 1.526 | 0.01276 | 0.6298 | 1.642 | 0.6925 |
| 88 | 7.352 | 2.227 | 20.3 | 1.051 | 0.0087 | 1.3064 | 6.086 | 0.9334 |
| 89 | 5.989 | 1.172 | 18.08 | 0.465 | 0.00383 | 0.9334 | 3.154 | 0.9055 |
| 90 | 7.049 | 1.504 | 17.4 | 0.238 | 0.00192 | 1.093 | 4.184 | 0.9675 |
| 91 | 7.422 | 1.921 | 18.68 | 0.779 | 0.00643 | 1.2088 | 5.23 | 0.9263 |
| 92 | 7.633 | 4.173 | 26.76 | 4.153 | 0.03461 | 1.787 | 11.395 | 0.9319 |
| 93 | 6.899 | 1.972 | 20.36 | 1.073 | 0.0089 | 1.1993 | 5.257 | 0.8884 |
| 94 | 7.32 | 2.228 | 20.39 | 1.129 | 0.00935 | 1.299 | 6.053 | 0.9222 |
| 95 | 7.366 | 2.482 | 21.39 | 2.549 | 0.02125 | 1.2574 | 6.183 | 0.7757 |
| 96 | 7.429 | 3.027 | 23.42 | 2.366 | 0.01969 | 1.4998 | 8.145 | 0.9051 |
| 97 | 7.303 | 1.936 | 19.05 | 3.306 | 0.02762 | 0.9557 | 4.151 | 0.5744 |
| 98 | 7.656 | 1.553 | 16.28 | 1.042 | 0.00866 | 1.0307 | 4.01 | 0.833 |
| 99 | 6.751 | 1.903 | 20.43 | 0.474 | 0.00387 | 1.2326 | 5.308 | 0.9724 |
| 101 | 7.057 | 1.666 | 18.29 | 1.567 | 0.01305 | 1.0233 | 4.124 | 0.7654 |
| 102 | 7.332 | 0.869 | 12.71 | 0.262 | 0.00215 | 0.8298 | 2.414 | 0.9654 |
| 103 | 7.413 | 1.09 | 14.09 | 2.689 | 0.02249 | 0.6716 | 2.189 | 0.5039 |
| 104 | 7.512 | 2.041 | 19.02 | 0.882 | 0.00729 | 1.2472 | 5.562 | 0.9284 |
| 105 | 7.412 | 2.851 | 22.78 | 2.691 | 0.02241 | 1.3943 | 7.349 | 0.8305 |
| 106 | 7.948 | 3.806 | 24.55 | 3.71 | 0.03091 | 1.6817 | 10.241 | 0.905 |
| 107 | 7.475 | 1.469 | 16.21 | 1.55 | 0.01292 | 0.9439 | 3.571 | 0.7388 |
| 108 | 7.334 | 1.182 | 14.82 | 1.389 | 0.01159 | 0.8426 | 2.859 | 0.7316 |
| 109 | 7.209 | 2.319 | 21.12 | 1.65 | 0.01372 | 1.2826 | 6.096 | 0.864 |
| 110 | 7.958 | 1.58 | 15.8 | 1.668 | 0.01391 | 0.9776 | 3.836 | 0.7365 |
| 111 | 7.4 | 0.445 | 9.02 | 0.873 | 0.00729 | 0.5933 | 1.236 | 0.9626 |
| 112 | 7.376 | 1.321 | 15.58 | 0.237 | 0.00191 | 1.0178 | 3.651 | 0.9555 |
| 113 | 7.013 | 0.783 | 12.62 | 2.393 | 0.02002 | 0.5758 | 1.59 | 0.5158 |
| 114 | 7.714 | 0.762 | 11.32 | 1.514 | 0.01266 | 0.6574 | 1.791 | 0.6908 |
| 115 | 5.927 | 2.263 | 25.38 | 6.191 | 0.0518 | 0.7937 | 3.727 | 0.339 |
| 116 | 6.806 | 1.661 | 18.93 | 3.04 | 0.0254 | 0.8698 | 3.499 | 0.5549 |
| 117 | 6.905 | 0.712 | 12.22 | 0.585 | 0.00487 | 0.7321 | 1.928 | 0.9174 |
| 118 | 7.663 | 1.25 | 14.59 | 1.464 | 0.01221 | 0.8628 | 3.011 | 0.7255 |
| 119 | 6.647 | 1.295 | 17.12 | 0.676 | 0.0056 | 0.9621 | 3.417 | 0.8707 |
| 121 | 6.243 | 0.628 | 12.69 | 0.809 | 0.00675 | 0.6747 | 1.669 | 0.883 |
| 122 | 6.959 | 1.096 | 15.05 | 0.064 | 0.00048 | 0.9435 | 3.084 | 0.9889 |
| 123 | 6.814 | 1.348 | 17.04 | 0.587 | 0.00485 | 0.9933 | 3.6 | 0.8912 |
| 124 | 6.539 | 1.785 | 20.43 | 0.903 | 0.00748 | 1.14 | 4.754 | 0.8869 |
| 125 | 7.811 | 2.872 | 21.7 | 1.769 | 0.01469 | 1.4975 | 7.922 | 0.951 |
| 126 | 7.38 | 2.952 | 23.28 | 2.345 | 0.01951 | 1.4713 | 7.891 | 0.8931 |
| 127 | 7.805 | 2.356 | 19.67 | 0.964 | 0.00796 | 1.3682 | 6.556 | 0.9678 |
| 128 | 6.751 | 2.847 | 24.99 | 2.377 | 0.01979 | 1.4247 | 7.503 | 0.8684 |
| 129 | 6.764 | 1.03 | 15.01 | 0.457 | 0.00377 | 0.8762 | 2.776 | 0.9074 |
| 130 | 6.806 | 0.81 | 13.22 | 0.482 | 0.004 | 0.7829 | 2.199 | 0.922 |
| 131 | 6.989 | 2.905 | 24.39 | 1.486 | 0.01231 | 1.5401 | 8.194 | 0.9944 |
| 132 | 7.183 | 1.481 | 16.94 | 2.187 | 0.01826 | 0.8836 | 3.357 | 0.642 |
| 133 | 6.501 | 1.251 | 17.21 | 2.656 | 0.0222 | 0.7412 | 2.588 | 0.5347 |
| 134 | 6.968 | 1.17 | 15.53 | 0.454 | 0.00375 | 0.9339 | 3.154 | 0.9076 |
| 135 | 6.933 | 1.764 | 19.16 | 2.329 | 0.01944 | 0.9851 | 4.084 | 0.67 |
| 136 | 6.954 | 3.722 | 27.74 | 3.825 | 0.03188 | 1.6355 | 9.849 | 0.8752 |
| 137 | 7.148 | 2.826 | 23.52 | 1.629 | 0.01351 | 1.4928 | 7.833 | 0.9605 |
| 138 | 6.814 | 4.568 | 31.37 | 7.706 | 0.06438 | 1.5845 | 10.571 | 0.6694 |
| 139 | 7.329 | 4.328 | 28.38 | 5.065 | 0.04225 | 1.7569 | 11.409 | 0.8687 |
| 141 | 5.937 | 1.535 | 20.87 | 0.448 | 0.00367 | 1.0841 | 4.192 | 0.9327 |
| 142 | 7.093 | 1.721 | 18.5 | 0.239 | 0.00191 | 1.1822 | 4.841 | 0.9888 |
| 143 | 6.739 | 1.498 | 18.16 | 0.475 | 0.00391 | 1.066 | 4.072 | 0.9242 |
| 144 | 6.995 | 0.552 | 10.62 | 1.231 | 0.01029 | 0.6002 | 1.392 | 0.7952 |
| 145 | 6.198 | 1.429 | 19.29 | 1.184 | 0.00985 | 0.9649 | 3.6 | 0.7938 |
| 146 | 5.929 | 0.859 | 15.63 | 2.816 | 0.02357 | 0.5634 | 1.63 | 0.4504 |
| 147 | 5.764 | 1.362 | 20.25 | 1.871 | 0.01562 | 0.8672 | 3.16 | 0.6724 |
| 148 | 6.427 | 2.199 | 23.08 | 4.423 | 0.03697 | 0.9489 | 4.393 | 0.4987 |
| 149 | 6.45 | 1.941 | 21.6 | 0.525 | 0.0043 | 1.2428 | 5.404 | 0.9693 |
| 150 | 6.924 | 1.1 | 15.15 | 3.376 | 0.02825 | 0.6051 | 1.981 | 0.4054 |
| 151 | 7.349 | 0.757 | 11.84 | 2.933 | 0.02456 | 0.5096 | 1.384 | 0.418 |
| 152 | 6.928 | 0.511 | 10.32 | 0.965 | 0.00806 | 0.611 | 1.364 | 0.8889 |
| 153 | 7.367 | 0.765 | 11.87 | 5.99 | 0.05019 | 0.1993 | 0.544 | 0.0632 |
| 154 | 6.832 | 1.105 | 15.39 | 0.93 | 0.00774 | 0.8582 | 2.816 | 0.8118 |
| 155 | 8.319 | 1.667 | 15.52 | 1.469 | 0.01223 | 1.0336 | 4.166 | 0.7805 |
| 156 | 7.289 | 1.994 | 19.37 | 3.11 | 0.02597 | 0.9995 | 4.406 | 0.6101 |
| 157 | 7.101 | 1.556 | 17.57 | 2.429 | 0.02028 | 0.8896 | 3.464 | 0.6194 |
| 158 | 7.588 | 0.902 | 12.52 | 0.303 | 0.00249 | 0.8393 | 2.488 | 0.9509 |
| 159 | 7.002 | 1.311 | 16.35 | 0.371 | 0.00304 | 1.0003 | 3.576 | 0.9292 |
| 161 | 7.635 | 2.668 | 21.39 | 2.43 | 0.02024 | 1.3457 | 6.861 | 0.8268 |
| 162 | 6.988 | 1.631 | 18.28 | 0.975 | 0.00809 | 1.0695 | 4.263 | 0.8541 |
| 163 | 6.775 | 0.77 | 12.95 | 1.014 | 0.00846 | 0.7121 | 1.951 | 0.8019 |
| 164 | 6.927 | 1.035 | 14.69 | 0.185 | 0.00149 | 0.9059 | 2.877 | 0.9658 |
| 165 | 6.173 | 1.011 | 16.29 | 0.595 | 0.00494 | 0.8538 | 2.679 | 0.8785 |
| RS2: Ranked Environmental Variance; RCV: Ranked Environmental Coefficient of Variation; RW2: Ranked Shukla Environmental Variance; Rsig2: Ranked Significance of Regression Model; Rb: Ranked Coefficient of Regression Lines; Rsd: Ranked Standard Deviation or Error Term in Regression Model; RR2: Ranked Coefficient of Determination of the regression model. | | | | | | | | |
|  | | | | | | | | |
