# Supplementary material for: Evaluating multiple stability methods to screen bread wheat genotypes (F7 generation) under drought-stressed environments
Source: PeerJ. 2026 Feb 23;14:e20505. doi: 10.7717/peerj.20505 (PMC12939790; doi:10.7717/peerj.20505)
Supplement: Supplemental Information 5 [file peerj-14-20505-s005.docx]

| Supplementary Table 4. Rank of each univariate stability index for each genotype across all environments. | | | | | | | |
| --- | --- | --- | --- | --- | --- | --- | --- |
| Genotype | RS2 | RCV | RW2 | Rsig2 | Rb | Rsd | RR2 |
| 1 | 75 | 127 | 136 | 137 | 41 | 55 | 20 |
| 2 | 105 | 106 | 116 | 116 | 97 | 103 | 47 |
| 3 | 30 | 28 | 12 | 12 | 49 | 39 | 139 |
| 4 | 106 | 105 | 151 | 151 | 39 | 65 | 7 |
| 5 | 10 | 9 | 47 | 47 | 23 | 21 | 91 |
| 6 | 3 | 2 | 69 | 69 | 14 | 6 | 74 |
| 7 | 45 | 46 | 36 | 36 | 58 | 50 | 95 |
| 8 | 86 | 103 | 96 | 96 | 87 | 88 | 53 |
| 9 | 123 | 117 | 92 | 92 | 122 | 123 | 81 |
| 10 | 108 | 84 | 70 | 70 | 113 | 112 | 90 |
| 11 | 97 | 89 | 89 | 89 | 105 | 104 | 66 |
| 12 | 82 | 75 | 160 | 160 | 3 | 8 | 3 |
| 13 | 24 | 25 | 123 | 124 | 11 | 17 | 15 |
| 14 | 1 | 3 | 120 | 120 | 4 | 2 | 21 |
| 15 | 22 | 31 | 130 | 130 | 8 | 15 | 10 |
| 16 | 78 | 65 | 15 | 15 | 104 | 97 | 138 |
| 17 | 95 | 104 | 135 | 135 | 65 | 83 | 27 |
| 18 | 11 | 6 | 109 | 109 | 9 | 9 | 23 |
| 19 | 93 | 122 | 161 | 161 | 2 | 4 | 1 |
| Danesh | 14 | 10 | 121 | 121 | 7 | 12 | 13 |
| 21 | 8 | 12 | 72 | 73 | 16 | 14 | 55 |
| 22 | 7 | 11 | 141 | 141 | 5 | 3 | 4 |
| 23 | 39 | 53 | 100 | 101 | 28 | 34 | 33 |
| 24 | 114 | 129 | 102 | 102 | 108 | 110 | 60 |
| 25 | 15 | 17 | 10 | 10 | 36 | 28 | 161 |
| 26 | 57 | 58 | 6 | 6 | 82 | 64 | 144 |
| 27 | 112 | 94 | 23 | 23 | 131 | 127 | 154 |
| 28 | 118 | 142 | 24 | 24 | 138 | 131 | 158 |
| 29 | 142 | 152 | 101 | 100 | 145 | 143 | 116 |
| 30 | 137 | 148 | 98 | 98 | 135 | 138 | 89 |
| 31 | 151 | 150 | 157 | 157 | 109 | 140 | 18 |
| 32 | 132 | 136 | 73 | 72 | 137 | 136 | 114 |
| 33 | 138 | 133 | 158 | 158 | 43 | 84 | 5 |
| 34 | 129 | 145 | 35 | 34 | 140 | 139 | 155 |
| 35 | 102 | 134 | 60 | 60 | 115 | 111 | 103 |
| 36 | 81 | 108 | 110 | 110 | 64 | 73 | 36 |
| 37 | 110 | 118 | 63 | 63 | 118 | 117 | 102 |
| 38 | 34 | 68 | 41 | 41 | 44 | 40 | 83 |
| 39 | 148 | 160 | 137 | 136 | 143 | 145 | 67 |
| Amin | 69 | 40 | 26 | 26 | 94 | 82 | 122 |
| 41 | 155 | 159 | 154 | 154 | 147 | 153 | 39 |
| 42 | 121 | 111 | 86 | 86 | 120 | 121 | 86 |
| 43 | 89 | 90 | 104 | 104 | 80 | 85 | 48 |
| 44 | 83 | 123 | 108 | 108 | 68 | 76 | 38 |
| 45 | 53 | 60 | 61 | 61 | 56 | 53 | 65 |
| 46 | 5 | 15 | 74 | 74 | 13 | 7 | 59 |
| 47 | 44 | 36 | 83 | 83 | 40 | 41 | 43 |
| 48 | 56 | 80 | 1 | 1 | 86 | 67 | 160 |
| 49 | 29 | 34 | 67 | 67 | 31 | 32 | 58 |
| 50 | 63 | 72 | 3 | 3 | 96 | 79 | 152 |
| 51 | 32 | 35 | 68 | 68 | 35 | 35 | 57 |
| 52 | 33 | 30 | 77 | 78 | 32 | 33 | 49 |
| 53 | 23 | 18 | 33 | 33 | 34 | 29 | 105 |
| 54 | 141 | 132 | 149 | 148 | 111 | 132 | 30 |
| 55 | 19 | 7 | 122 | 122 | 10 | 16 | 14 |
| 56 | 31 | 26 | 103 | 103 | 26 | 27 | 29 |
| 57 | 133 | 110 | 125 | 125 | 114 | 125 | 51 |
| 58 | 96 | 78 | 84 | 84 | 106 | 105 | 68 |
| 59 | 116 | 87 | 91 | 90 | 112 | 113 | 77 |
| Farin | 157 | 156 | 146 | 146 | 157 | 157 | 93 |
| 61 | 120 | 98 | 93 | 93 | 117 | 120 | 79 |
| 62 | 119 | 85 | 124 | 123 | 102 | 109 | 44 |
| 63 | 48 | 55 | 99 | 99 | 37 | 44 | 35 |
| 64 | 98 | 96 | 139 | 139 | 71 | 87 | 26 |
| 65 | 124 | 121 | 57 | 56 | 133 | 130 | 126 |
| 66 | 67 | 77 | 38 | 38 | 89 | 77 | 100 |
| 67 | 42 | 37 | 27 | 27 | 61 | 51 | 109 |
| 68 | 100 | 102 | 88 | 88 | 107 | 107 | 69 |
| 69 | 55 | 42 | 37 | 37 | 67 | 60 | 94 |
| 70 | 65 | 54 | 28 | 28 | 88 | 74 | 117 |
| 71 | 113 | 74 | 44 | 44 | 127 | 122 | 127 |
| 72 | 117 | 153 | 147 | 147 | 62 | 89 | 16 |
| 73 | 152 | 143 | 142 | 142 | 152 | 152 | 80 |
| 74 | 91 | 71 | 66 | 66 | 100 | 102 | 82 |
| 75 | 131 | 119 | 106 | 106 | 125 | 128 | 71 |
| 76 | 158 | 154 | 152 | 152 | 155 | 156 | 63 |
| 77 | 47 | 29 | 105 | 105 | 33 | 38 | 31 |
| 78 | 136 | 144 | 153 | 153 | 66 | 101 | 11 |
| 79 | 122 | 114 | 49 | 49 | 132 | 129 | 134 |
| Torabi | 70 | 64 | 32 | 32 | 92 | 81 | 110 |
| 81 | 125 | 131 | 94 | 94 | 124 | 126 | 85 |
| 82 | 109 | 100 | 59 | 59 | 121 | 116 | 108 |
| 83 | 54 | 43 | 4 | 4 | 78 | 61 | 148 |
| 84 | 36 | 32 | 46 | 46 | 45 | 43 | 78 |
| 85 | 153 | 141 | 133 | 133 | 156 | 154 | 124 |
| 86 | 49 | 57 | 56 | 57 | 55 | 52 | 70 |
| 87 | 12 | 21 | 80 | 80 | 21 | 20 | 46 |
| 88 | 127 | 109 | 55 | 55 | 139 | 134 | 137 |
| 89 | 51 | 79 | 19 | 19 | 69 | 58 | 120 |
| 90 | 74 | 73 | 8 | 9 | 103 | 95 | 149 |
| 91 | 101 | 88 | 39 | 39 | 123 | 114 | 131 |
| 92 | 159 | 155 | 145 | 145 | 161 | 160 | 135 |
| 93 | 107 | 112 | 58 | 58 | 119 | 115 | 111 |
| 94 | 128 | 113 | 62 | 62 | 136 | 133 | 129 |
| 95 | 139 | 125 | 119 | 119 | 130 | 137 | 62 |
| 96 | 150 | 139 | 113 | 113 | 151 | 150 | 119 |
| 97 | 103 | 93 | 138 | 138 | 75 | 93 | 28 |
| 98 | 77 | 61 | 54 | 54 | 93 | 86 | 88 |
| 99 | 99 | 116 | 20 | 20 | 126 | 118 | 153 |
| 101 | 87 | 83 | 82 | 82 | 91 | 92 | 61 |
| 102 | 27 | 23 | 11 | 11 | 46 | 36 | 146 |
| 103 | 40 | 33 | 127 | 128 | 24 | 30 | 19 |
| 104 | 115 | 92 | 43 | 42 | 129 | 124 | 132 |
| 105 | 145 | 135 | 128 | 127 | 144 | 144 | 87 |
| 106 | 156 | 147 | 143 | 143 | 159 | 158 | 118 |
| 107 | 71 | 59 | 81 | 81 | 73 | 69 | 56 |
| 108 | 52 | 41 | 71 | 71 | 48 | 48 | 52 |
| 109 | 134 | 124 | 87 | 87 | 134 | 135 | 96 |
| 110 | 80 | 56 | 90 | 91 | 79 | 80 | 54 |
| 111 | 2 | 1 | 42 | 43 | 17 | 5 | 145 |
| 112 | 62 | 51 | 7 | 8 | 90 | 75 | 142 |
| 113 | 21 | 20 | 115 | 115 | 15 | 18 | 22 |
| 114 | 17 | 8 | 79 | 79 | 22 | 23 | 45 |
| 115 | 130 | 151 | 156 | 156 | 42 | 78 | 6 |
| 116 | 85 | 91 | 132 | 132 | 54 | 68 | 25 |
| 117 | 13 | 16 | 29 | 30 | 29 | 24 | 125 |
| 118 | 58 | 38 | 75 | 75 | 52 | 54 | 50 |
| 119 | 60 | 69 | 34 | 35 | 76 | 63 | 99 |
| 121 | 9 | 22 | 40 | 40 | 25 | 22 | 106 |
| 122 | 41 | 45 | 2 | 2 | 72 | 56 | 157 |
| 123 | 64 | 67 | 30 | 29 | 83 | 72 | 113 |
| 124 | 94 | 115 | 45 | 45 | 110 | 106 | 107 |
| 125 | 146 | 130 | 95 | 95 | 150 | 149 | 141 |
| 126 | 149 | 138 | 112 | 112 | 148 | 148 | 115 |
| 127 | 135 | 101 | 50 | 50 | 142 | 141 | 150 |
| 128 | 144 | 149 | 114 | 114 | 146 | 146 | 97 |
| 129 | 37 | 44 | 18 | 18 | 57 | 46 | 121 |
| 130 | 25 | 27 | 22 | 22 | 38 | 31 | 128 |
| 131 | 147 | 146 | 78 | 77 | 153 | 151 | 159 |
| 132 | 72 | 66 | 107 | 107 | 59 | 62 | 37 |
| 133 | 59 | 70 | 126 | 126 | 30 | 42 | 24 |
| 134 | 50 | 50 | 17 | 17 | 70 | 57 | 123 |
| 135 | 92 | 95 | 111 | 111 | 81 | 91 | 41 |
| 136 | 154 | 157 | 144 | 144 | 158 | 155 | 101 |
| 137 | 143 | 140 | 85 | 85 | 149 | 147 | 143 |
| 138 | 161 | 161 | 159 | 159 | 154 | 159 | 40 |
| 139 | 160 | 158 | 150 | 150 | 160 | 161 | 98 |
| 141 | 76 | 120 | 16 | 16 | 101 | 96 | 136 |
| 142 | 90 | 86 | 9 | 7 | 116 | 108 | 156 |
| 143 | 73 | 81 | 21 | 21 | 98 | 90 | 130 |
| 144 | 6 | 5 | 65 | 65 | 18 | 13 | 73 |
| 145 | 68 | 97 | 64 | 64 | 77 | 71 | 72 |
| 146 | 26 | 52 | 129 | 129 | 12 | 19 | 12 |
| 147 | 66 | 107 | 97 | 97 | 53 | 59 | 42 |
| 148 | 126 | 137 | 148 | 149 | 74 | 99 | 17 |
| 149 | 104 | 128 | 25 | 25 | 128 | 119 | 151 |
| 150 | 43 | 47 | 140 | 140 | 19 | 26 | 8 |
| 151 | 16 | 13 | 131 | 131 | 6 | 11 | 9 |
| 152 | 4 | 4 | 51 | 51 | 20 | 10 | 112 |
| 153 | 18 | 14 | 155 | 155 | 1 | 1 | 2 |
| 154 | 46 | 48 | 48 | 48 | 51 | 47 | 76 |
| 155 | 88 | 49 | 76 | 76 | 95 | 94 | 64 |
| 156 | 111 | 99 | 134 | 134 | 84 | 100 | 32 |
| 157 | 79 | 76 | 117 | 118 | 60 | 66 | 34 |
| 158 | 28 | 19 | 13 | 13 | 47 | 37 | 140 |
| 159 | 61 | 63 | 14 | 14 | 85 | 70 | 133 |
| 161 | 140 | 126 | 118 | 117 | 141 | 142 | 84 |
| 162 | 84 | 82 | 52 | 52 | 99 | 98 | 92 |
| 163 | 20 | 24 | 53 | 53 | 27 | 25 | 75 |
| 164 | 38 | 39 | 5 | 5 | 63 | 49 | 147 |
| 165 | 35 | 62 | 31 | 31 | 50 | 45 | 104 |
| RS2 RCV RW2 Rsig2 Rb Rsd RR2 | | | | | | | |
