# Supplementary material for: Evaluating multiple stability methods to screen bread wheat genotypes (F7 generation) under drought-stressed environments
Source: PeerJ. 2026 Feb 23;14:e20505. doi: 10.7717/peerj.20505 (PMC12939790; doi:10.7717/peerj.20505)
Supplement: Supplemental Information 6 [file peerj-14-20505-s006.docx]

| 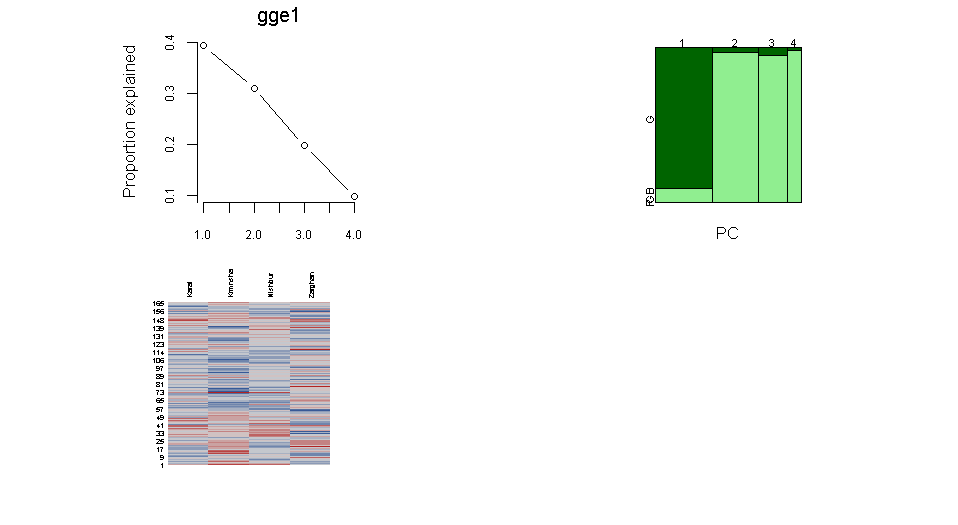 | 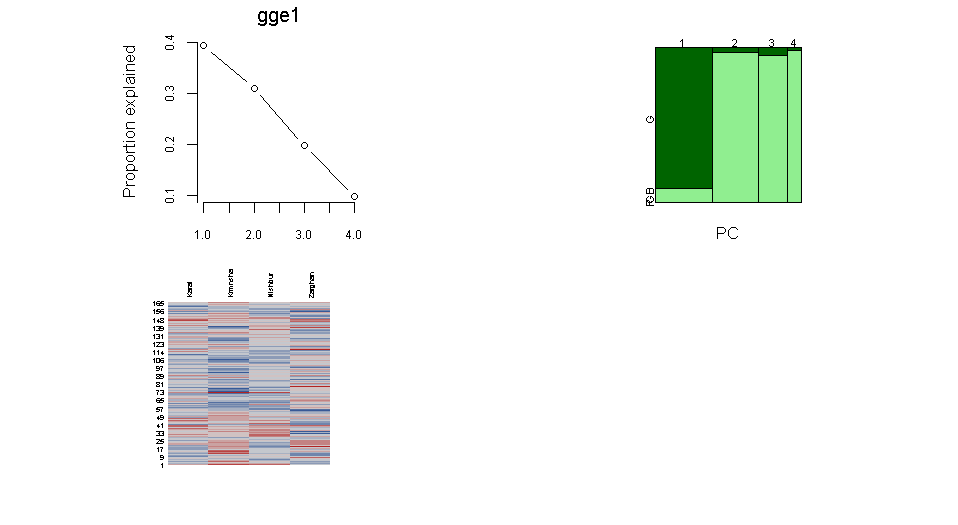 |
| --- | --- |
| Supplementary Figure 1. The proportion of each component in genotype plus genotype by environment analysis. | |
